# The impact of fish oil and/or probiotics on serum fatty acids and the interaction with low-grade inflammation in pregnant women with overweight and obesity: secondary analysis of a randomised controlled trial

**DOI:** 10.1017/S0007114523001915

**Published:** 2024-01-28

**Authors:** Noora Houttu, Tero Vahlberg, Elizabeth A. Miles, Philip C. Calder, Kirsi Laitinen

**Affiliations:** 1 Institute of Biomedicine, Integrative Physiology and Pharmacology Unit, University of Turku, 20520 Turku, Finland; 2 Department of Clinical Medicine, Biostatistics, University of Turku, 20520 Turku, Finland; 3 School of Human Development and Health, Faculty of Medicine, University of Southampton, Southampton SO16 6YD, UK; 4 NIHR Southampton Biomedical Research Centre, University Hospital Southampton NHS Foundation Trust and University of Southampton, Southampton SO16 6YD, UK; 5 Department of Obstetrics and Gynaecology, Turku University Hospital, 20500 Turku, Finland; 6 Functional Foods Forum, University of Turku, Turku, Finland

**Keywords:** Fish oil, Probiotics, Serum fatty acids, Low-grade inflammation, Gestational diabetes mellitus

## Abstract

*N*-3 long-chain PUFA (LC-PUFA) and probiotics are generally considered to induce health benefits. The objective was to investigate (1) the impact of fish oil and/or probiotics on serum fatty acids (sFA), (2) the interaction of sFA with low-grade inflammation and (3) the relation of sFA to the onset of gestational diabetes mellitus (GDM). Pregnant women with overweight/obesity were allocated into intervention groups with fish oil + placebo, probiotics + placebo, fish oil + probiotics or placebo + placebo in early pregnancy (fish oil: 1·9 g DHA and 0·22 g EPA, probiotics: *Lacticaseibacillus rhamnosus* HN001 and *Bifidobacterium animalis* ssp*. lactis* 420, 10^10^ CFU, each daily). Blood samples were collected in early (*n* 431) and late pregnancy (*n* 361) for analysis of fatty acids in serum phosphatidylcholine (PC), cholesteryl esters (CE), TAG and NEFA with GC and high-sensitivity C-reactive protein and GlycA by immunoassay and NMR spectroscopy, respectively. GDM was diagnosed according to 2 h 75 g oral glucose tolerance test. EPA in PC, CE and TAG and DHA in PC, CE, TAG and NEFA were higher in fish oil and fish oil + probiotics groups compared with placebo. EPA in serum NEFA was lower in women receiving probiotics compared with women not receiving. Low-grade inflammation was inversely associated with *n*-3 LC-PUFA, which were related to an increased risk of GDM. Fish oil and fish oil + probiotics consumption increase serum *n*-3 LC-PUFA in pregnant women with overweight/obesity. Although these fatty acids were inversely related to inflammatory markers, *n*-3 LC-PUFA were linked with an increased risk for GDM.

Long-chain PUFA (LC-PUFA) are transferred from the mother to the fetus during pregnancy to meet the fetal needs for development; maternal LC-PUFA supply is crucial since the synthesis of LC-PUFA by the fetus and placenta is very low^(1)^. In particular, *n*-3 LC-PUFA are critical for the child’s neurodevelopment^(2–4)^ and may lower the risk of allergic diseases^(5)^. Additionally, recent studies highlight the importance of *n*-3 and *n*-6 LC-PUFA status to maternal metabolic health, as fatty acid levels have been linked with gestational diabetes mellitus (GDM), although the findings are somewhat inconsistent: a higher concentration of plasma total *n*-3 LC-PUFA and *n*-6 LC-PUFA and a lower percentage of plasma total *n*-6 LC-PUFA were positively related to the onset of GDM^(6)^ whereas in another study, a lower percentage of erythrocyte *n*-3 LC-PUFA and a higher percentage of erythrocyte *n*-6 LC-PUFA were detected in women with GDM compared with women without GDM^(7)^.

Previous evidence has indicated that consumption of fish oil which is rich in *n*-3 LC-PUFA, primarily DHA and EPA, during pregnancy increases *n*-3 LC-PUFA levels in maternal blood^(8)^. In the blood, *n*-3 and *n*-6 LC-PUFA and other fatty acids are either esterified as phospholipids (PL) (e.g. phosphatidylcholine (PC)), cholesteryl esters (CE) and TAG or bound to albumin in the form of NEFA. Although techniques to measure fatty acids in all four lipid fractions are available, many of the prior studies of pregnant women have utilised the PC fraction to analyse blood fatty acids^(8–10)^.

Interestingly, preliminary evidence shows that probiotics may affect serum fatty acid (sFA) levels: the administration of *Lactobacillus gasseri* SBT2055 decreased total serum NEFA levels in adults with hypertriacylglycerolaemia^(11)^ and dietary intervention with counselling plus *Lacticaseibacillus rhamnosus* GG and *Bifidobacterium lactis* Bb12 (diet/probiotic group) increased *α*-linolenic acid and total *n*-3 fatty acids in breast milk compared with the control group (control/placebo) and increased *γ*-linolenic acid compared with the placebo group (diet/placebo)^(12)^.

Less is known about the combination of fish oil and probiotics. In our previous study in pregnant women, the combination of fish oil and probiotics increased serum DHA and *n*-3 fatty acids as well as the ratio of PUFA to total fatty acids as measured by NMR^(13)^. It is of note that in that study, fish oil and/or probiotics supplementation from early pregnancy onwards did not have an effect on the incidence of GDM^(14)^. Furthermore, Zhou *et al*.^(15)^ found that fish oil in pregnancy did not affect the risk of GDM. Regarding the effect of probiotics, previous studies have shown that they may improve glucose metabolism^(16)^ and reduce the risk of GDM^(17)^. An analysis of LC-PUFA in serum might provide insight into the impacts of fish oil and probiotics on fatty acids and subsequently on GDM risk, the proposed mechanism being modulation of low-grade inflammation.

We hypothesise that probiotics and fish oil jointly modify blood *n*-3 LC-PUFA levels above the effect induced with fish oil alone and that the *n*-3 LC-PUFA are related to reduced low-grade inflammation and the onset of GDM in pregnant women with overweight/obesity. The objective of this study was to (1) investigate the impact of the fish oil and probiotics separately and in combination compared with placebo on sFA, particularly *n*-3 LC-PUFA levels in four lipid fractions (PC, NEFA, CE and TAG), (2) study the interaction of serum *n*-3 LC-PUFA and low-grade inflammation (high-sensitivity C-reactive protein (hsCRP) and GlycA) and (3) investigate whether serum *n*-3 and also *n*-6 LC-PUFA are related to the onset of GDM in pregnant women with overweight and obesity.

## Materials and methods

### Study design and participants

**Fig. 1.**
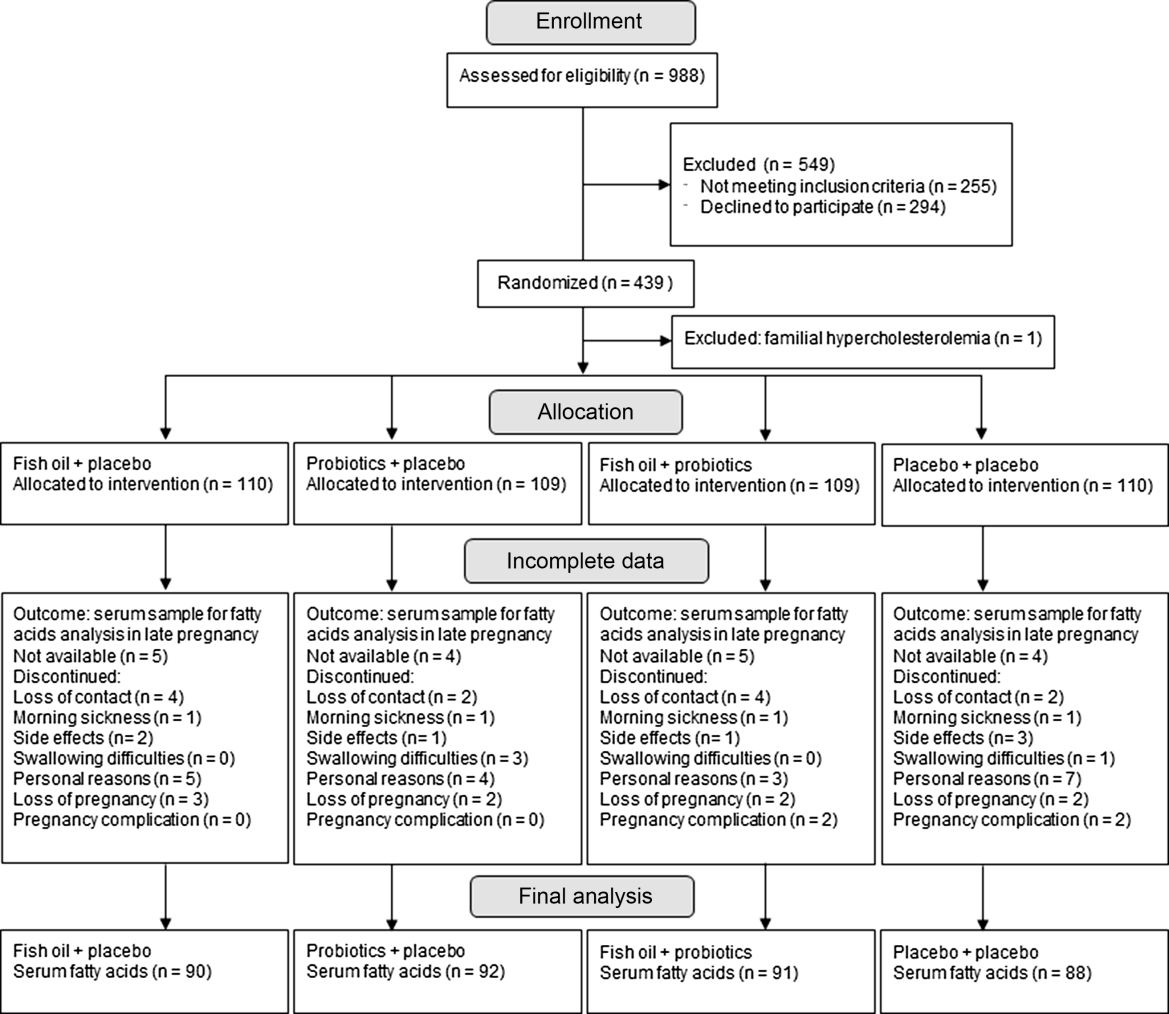
Flow diagram of the study.

This single centre double-blind, placebo-controlled randomised trial^(14)^ was conducted in the Turku University Hospital and University of Turku in Finland with recruitment between October 2013 and July 2017 (ClinicalTrials.gov, NCT01922791). The study complies with the Declaration of Helsinki as revised in 2000. The Ethics Committee of the Hospital District of Southwest Finland approved the study protocol, and all participants provided written informed consent. Pregnant women with overweight or obesity were randomised into four intervention groups at the first study visit during early pregnancy: fish oil + placebo, probiotics + placebo, fish oil + probiotics or placebo + placebo. A total of 439 women were recruited from Southwest Finland (Fig. 1, flow diagram). The inclusion criteria were overweight or obesity (self-reported pre-pregnancy BMI ≥ 25 kg/m^2^), early pregnancy (< 18 gestational weeks) and no chronic diseases (asthma and allergies were allowed).

For this secondary analysis of the main trial, blood samples were obtained at two study visits, early and late pregnancy (mean 13·8 (sd 2·1) and 35·2 (sd 1·0) gestational weeks, respectively). The inclusion criterion for this sub-study was the availability of blood samples for fatty acid analyses. There were 431 samples available in early pregnancy and 361 samples in late pregnancy. In the analysis evaluating the relation between serum *n*-3 LC-PUFA and low-grade inflammation, the women who had reported having any infection (*n* 50 in early pregnancy, *n* 43 in late pregnancy) and using antibiotics within 2 weeks before sampling (*n* 23 in early pregnancy, *n* 10 in late pregnancy) were excluded.

### Clinical parameters

Weight was obtained from welfare women clinic records and was self-reported. Height was measured with a wall stadiometer at the first visit. Pre-pregnancy BMI (kg/m^2^) was calculated by dividing weight in kilograms by height in metres squared. Overweight was defined as BMI ≥ 25 < 30 kg/m^2^ and obesity as BMI ≥ 30 kg/m^2^.

GDM was diagnosed at 24–28 gestational weeks on the basis of a 2 h 75 g oral glucose tolerance test if one or more values were: 0 h ≥ 5·3, 1 h ≥ 10·0 and 2 h ≥ 8·6 mmol/l^(18)^.

Blood pressure was measured with an Omron M5-1 (Intelli^TM^ sense, Omron Matsusaka Co., Ltd) on the left arm.

Three-day food diaries were obtained in early pregnancy. The daily intakes of energy and energy-yielding nutrients and fibre were calculated by using computerised software (AivoDiet 2.0.2.3, Aivo) utilising the Finnish Food Composition Database Fineli^(19)^.

Questionnaires were collected to obtain information about education and smoking, and the participants were interviewed about their fish oil supplement usage before participation.

### Dietary intervention

Women consumed two fish oil capsules and one probiotic capsule (or matched placebos) daily from early pregnancy/first study visit until 6 months postpartum; herein, we include data from the intervention from early until late pregnancy. The fish oil capsules (Incromega E1070, Croda Europe Ltd) contained 2·4 g of *n*-3 LC-PUFA: 1·9 g DHA (22:6*n*-3), 0·22 g EPA (20:5*n*-3) and the remaining amount other *n*-3 fatty acids. The placebo capsules for fish oil consisted of 2·4 g medium-chain fatty acids (capric acid C8 54·6 % and caprylic acid C10 40·3 %). The fish oil and placebo capsules were of the same size, shape and colour and both had a lemon flavour. Probiotic capsules contained *Lacticaseibacillus rhamnosus* HN001 (formerly *Lactobacillus rhamnosus* HN001) (ATCC SD5675; DuPont) and *Bifidobacterium animalis* ssp*. lactis* 420 (DSM 22089; DuPont), each with 10^10^ colony-forming units per capsule. The placebo for the probiotics consisted of microcrystalline cellulose. The probiotic and placebo capsules were of the same size, shape and colour. The compliance to the intervention was good: 88·4 % as determined by interviewing, and 91·8 (sd 15·9) % as calculated from returned sample of fish oil capsules^(14)^, and the good compliance was confirmed in principal component analysis; a clear separation of the intervention groups according to lipids that reflected the intake of fish oil was detected previously^(13)^. The rationale of choosing fish oil rich in DHA and EPA was based on their inflammation-resolving capacity^(20)^ and important role in fetal development^(3)^ while *L. rhamnosus* HN001 is a well-characterised probiotic^(21)^ and *B. animalis* ssp. *lactis* 420 has shown to decrease inflammation in human studies^(22,23)^.

### Fatty acid analyses

Blood samples were obtained from the antecubital vein of the mother in the morning after at least 9 h fasting and were then separated into aliquots and frozen in −80°C. The fatty acid composition of four serum lipid fractions (PC, CE, TAG and NEFA) was determined by GC (Agilent Technologies). Samples were batch-analysed between 21 October 2018 and 15 February 2019. The methodology of these analyses is described in more detail in Fisk *et al*.^(24)^. Briefly, internal standards, dipentadecanoyl-PC, heneicosanoic acid, cholesteryl heptadecanoate and tripentadecanoin were added to each serum sample. Lipid was extracted into chloroform–methanol (2:1 vol/vol). PC, NEFA, CE and TAG were separated by solid-phase extraction on aminopropyl silica cartridges. Fatty acids were removed and simultaneously methylated to produce fatty acid methyl esters by heating in methanolic sulphuric acid. Fatty acid methyl esters were separated by GC and were identified by comparison with retention times of thirty-seven fatty acid methyl esters standards run alongside the samples. Finally, the fatty acid methyl esters were quantified using ChemStation software (Agilent Technologies) and Microsoft Excel (Microsoft Corporation). The data are expressed as concentration (μg/ml serum) and percentage of total fatty acids (%).

### Low-grade inflammation

Serum hsCRP was analysed using an automated colorimetric immunoassay on a Dade Behring Dimension RXL autoanalyzer (Siemens Healthcare) in a certified laboratory (TYKSLAB, the Hospital District of Southwest Finland). The lower limit of detection was 0·1 mg/l. The data are expressed as mg/l. A high-throughput proton NMR spectroscopy metabolomics platform (Nightingale) was used to quantify GlycA^(25)^. Data are expressed as mmol/l.

### Statistics

Natural log-transformation was performed for fatty acid variables with skewness > 1. Z-scores were calculated for all fatty acid variables. The effect of the intervention on the fatty acids was determined in late pregnancy: one-way ANOVA followed by Tukey’s post hoc test or Welch ANOVA followed by Tamhane’s T2 post hoc test. To control the baseline fatty acid data, the fatty acids, which differed between the intervention groups at late pregnancy, were evaluated with one-way ANOVA or Welch ANOVA at baseline, and thus no differences were detected. The fatty acid variables, which differed between the intervention groups at late pregnancy, were adjusted with their early pregnancy values in general linear model. To control the possible effect of smoking, the analyses, which showed difference in the fatty acids in late pregnancy between the intervention groups, were adjusted for smoking before pregnancy (yes/no). Further, those fatty acids, which were influenced by the smoking status, were further studied with independent-samples *t* test: they were compared between women who smoked before pregnancy and those who did not. Only the proportion of 18:1*n*-9 in serum TAG differed between women who smoked before pregnancy and those who did not, and thus the result of the intervention on the % of 18:1*n*-9 is presented with the adjustment. Groups of women receiving fish oil (fish oil + placebo and fish oil + probiotics) and the women not receiving fish oil (probiotics + placebo and placebo + placebo) and the women receiving probiotics (probiotics + placebo and fish oil + probiotics) and the women not receiving probiotics (fish oil + placebo and placebo + placebo) were combined, and two-way ANOVA was used for comparing the differences in *n*-3 fatty acids between women receiving probiotics or not receiving probiotics and women receiving fish oil and not receiving fish oil (fish oil *v*. no fish oil and probiotics *v*. no probiotics). The pregnancy-induced changes on the *n*-3 and *n*-6 LC-PUFA and their total amount were analysed in the placebo group. The change was calculated by subtracting the fatty acids in late pregnancy from early pregnancy. Total *n*-3 and *n*-6 LC-PUFA were calculated by summing the original variables of *n*-3 and *n*-6 LC-PUFA, respectively. The *n*-6/*n*-3 LC-PUFA ratio was calculated by dividing the total *n*-6 LC-PUFA by total *n*-3 LC-PUFA as measured as a concentration in each fraction. Associations between low-grade inflammatory markers, hsCRP and GlycA, and *n*-3 LC-PUFA in early and late pregnancy, separately, were tested with Pearson correlation. Logistic regression analyses were utilised in studying whether the *n*-3 and *n*-6 LC-PUFA are related to the onset of GDM. Univariate logistic regression was used for analysing the *n*-3 and *n*-6 fatty acids separately and multivariable logistic regression for combined sets of *n*-3 and *n*-6 fatty acids. In the multivariate logistic regression analysis, fatty acids, which were significant in the univariate logistic regression, were analysed. First, the correlations between the individual fatty acids were checked (two units, % and concentration, separately) and to avoid the intercorrelation (Pearson correlation coefficients > 0·7) fourteen models were constructed. The multivariable logistic regression was done with and without adjusting for intake of total fat and SFA in g separately. Logistic regression analyses were adjusted for intervention group. One-way ANOVA or Welch ANOVA was used for testing differences in normally distributed clinical characteristic variables while Kruskal–Wallis in non-normally distributed variables between the groups. Fisher’s exact and *χ* ^2^ tests were used for categorical clinical variables. The data are expressed as mean values and standard deviations, median (interquartile range (IQR), percentages (%), 95 % CI for mean change and Pearson *r* and OR and 95 % CI for OR. *P* < 0·05 is considered statistically significant.

## Results

### Baseline characteristics of the pregnant women

Clinical characteristics and diet intake of the women are presented in Table 1. Approximately 40 % of the women were living with obesity and the rest were overweight; women were highly educated with more than half having a college or university degree. No differences between the intervention groups were detected in the clinical parameters except the percentage of women smoking before pregnancy (*P* = 0·02, Table 1), which was highest (27·9 %) in the placebo group, which was considered in statistical analyses.

**Table 1.** The baseline characteristics and dietary intake of all pregnant women and according to the intervention groups (Mean values and standard deviations; inter-quartile ranges; numbers and percentages)

	Fish oil + placebo		Probiotics + placebo		Fish oil + probiotics		Placebo + placebo		*n*	*P*
Clinical parameters	Mean or Median or *n*	sd or IQR or %	Mean or Median or *n*	sd or IQR or %	Mean or Median or *n*	sd or IQR or %	Mean or Median or *n*	sd or IQR or %		
Age (years)	30·6	4·9	30·9	4·5	30·7	4·7	30·3	4·1	90/92/91/88	0·85*
College or university degree (*n*, %)	59	65·6	58	65·2	53	58·9	51	59·3	90/89/90/86	0·68†
Pre-pregnancy BMI (kg/m^2^)	29·4	27·2–33·0	28·0	26·5–30·7	28·3	25·8–31·4	29·3	26·3–32·2	90/92/91/88	0·10§
Obese‡ (*n*, %)	43	47·8	29	31·5	34	37·4	36	40·9	90/92/91/88	0·15†
GDM (*n*, %)	18	22·8	21	24·4	21	23·5	15	19·2	79/86/83/78	0·81†
Blood pressure, systolic (mmHg)	115·8	109·9–125·0	116·0	110·0–123·0	114·0	109·5–121·0	118·0	112·5–125·0	90/91/91/87	0·13§
Blood pressure, diastolic (mmHg)	77·9	8·5	75·9	9·0	75·8	7·3	76·3	7·8	90/91/91/87	0·27*
Smoking before pregnancy (*n*, %)	14	15·6	24	26·7	11	12·2	24	27·9	90/90/90/86	0·02†
Smoking during pregnancy (*n*, %)	1	1·1	5	5·6	4	4·5	4	7·0	90/89/89/86	0·23||
Used fish oil supplements before participation (*n*, %)	10	11·1	12	13·0	16	17·6	16	18·2	90/92/91/88	0·47†
Dietary intake										
Energy (kJ/d)	7759	1790	8077	2091	8322	2158	8424	1926	87/90/90/85	0·13*
Protein (E%/d)	16·2	14·3–18·3	16·8	14·2–18·4	16·5	14·9–18·5	17·2	14·8–19·2	87/90/90/85	0·44§
Carbohydrate (E%/d)	46·1	6·8	45·8	6·8	46·1	6·4	44·9	5·7	87/90/90/85	0·60*
Fat (E%/d)	35·0	6·8	35·2	6·6	34·6	6·1	35·4	6·7	87/90/90/85	0·87*
Fibre (g/d)	19·0	14·2–24·4	17·9	14·4–25·0	19·2	15·0–23·9	21·0	16·7–26·2	87/90/90/85	0·24§
SFA (E%/d)	12·4	3·3	12·9	3·0	12·7	2·8	13·0	3·1	87/90/90/85	0·60*
MUFA (E%/d)	12·0	2·9	12·2	2·7	11·8	2·7	12·3	3·0	87/90/90/85	0·62*
PUFA (E%/d)	5·8	1·9	5·6	1·6	5·5	1·3	5·5	1·5	87/90/90/85	0·53¶
*n*-3 fatty acids (E%/d)	1·6	1·3–1·9	1·5	1·3–1·8	1·6	1·3–1·8	1·5	1·4–1·8	87/90/90/85	0·54§
*α*-linolenic acid (g/d)	1·8	1·4–2·4	1·8	1·3–2·5	1·9	1·3–2·4	1·8	1·3–2·5	87/90/90/85	0·99§
EPA (mg/d)	18·0	6·1–94·3	15·5	8·0–52·4	16·3	8·8–107·0	18·0	11·0–72·7	87/90/90/85	0·46§
DHA (mg/d)	63·5	26·4–301·2	59·8	30·3–160·6	66·6	28·5–250·3	53·4	25·3–157·3	87/90/90/85	0·73§
*n*-6 fatty acids (E%/d)	4·3	3·4–5·2	4·1	3·4–5·0	4·1	3·3–4·8	4·2	3·5–5·0	87/90/90/85	0·66§
Linoleic acid (g/d)	7·2	5·0–9·8	7·0	5·2–10·1	6·8	5·5–9·3	7·2	5·4–9·6	87/90/90/85	0·98§

GDM, gestational diabetes mellitus.

The statistical significance is denoted with *P* < 0·05.

* One-way ANOVA.

§ Kruskal–Wallis.

¶ Welch ANOVA.

† *χ* ^2^.

‡ Obese is defined as BMI ≥ 30 kg/m^2^.

|| Fisher’s exact test.

### Change in *n*-3 and *n*-6 long-chain PUFA in the four lipid fractions during pregnancy

The pregnancy-induced changes in *n*-3 and *n*-6 LC-PUFA and total *n*-3 and *n*-6 LC-PUFA were evaluated in the placebo group (online Supplementary Table 1). Total *n*-3 LC-PUFA decreased in all four fractions, evaluated as both the % and concentration. EPA (20:5*n*-3) and DHA (22:6*n*-3) decreased from early to late pregnancy. Specifically, the % and the concentration of EPA (20:5*n*-3) decreased in serum PC, CE and TAG from early pregnancy to late pregnancy. The % and the concentration of DHA (22:6*n*-3) decreased in serum PC, NEFA and TAG.

With respect to *n*-6 LC-PUFA, the pregnancy-induced changes were seen only when fatty acids were expressed as %. The % of total *n*-6 LC-PUFA increased in serum PC and decreased in serum TAG. In more detail, the % of dihomo-*γ*-linolenic acid (DGLA, 20:3*n*-6) increased in serum PC from early to late pregnancy, while the % of linoleic acid (LA, 18:2*n*-6) decreased in serum TAG.

### Impact of the dietary intervention with fish oil and/or probiotics on serum fatty acid composition in the four lipid fractions

The dietary intervention had an impact on the total *n*-3 LC-PUFA in all four serum lipid fractions (PC, NEFA, CE and TAG in Tables 2–5, respectively). Specifically, the % and concentration of total *n*-3 LC-PUFA in the serum PC, NEFA, CE and TAG were statistically significantly higher in the fish oil and fish oil + probiotics groups compared with the probiotics and placebo groups (*P* < 0·001 for all comparisons). Regarding the specific *n*-3 LC-PUFA, the % and concentration of EPA (20:5*n*-3) were higher in the fish oil and fish oil + probiotics groups compared with the probiotics and placebo groups for serum PC, CE and TAG (*P* < 0·001 for all comparisons). In serum NEFA, the % and concentration of EPA (20:5*n*-3) were higher in the fish oil compared with the probiotics group (*P* = 0·001 and *P* = 0·003, respectively). The % and the concentration of DHA (22:6*n*-3) were higher in the fish oil and fish oil + probiotics groups compared with the probiotics and placebo groups in serum PC, NEFA, CE and TAG (*P* ≤ 0·001 for all comparisons). Additionally, docosapentaenoic acid (DPA, 22:5*n*-3) was higher in the fish oil and fish oil + probiotics groups compared with the probiotics and placebo groups in serum TAG (*P* ≤ 0·001 for all comparisons) when expressed as % and higher in the fish oil compared with the placebo group when expressed as concentration (*P* = 0·04).

**Table 2.** *n*-3 long-chain PUFA (LC-PUFA) in serum phosphatidylcholine (PC) as a percentage of total fatty acids (%) and absolute concentration (μg/ml) *z*-scores in pregnant women with overweight and obesity according to the four dietary intervention groups in late pregnancy (Mean values and standard deviations)

		PC fatty acids % of total (*z*-scores)					PC fatty acids absolute concentration (μg/ml) (*z*-scores)				
		Fish oil + placebo	Probiotics + placebo	Fish oil + probiotics	Placebo + placebo	*P*	Fish oil + placebo	Probiotics + placebo	Fish oil + probiotics	Placebo + placebo	*P*
n		90	91	91	88		90	91	91	88	
18:3*n*-3	Mean	–0·12	0·01	0·03	0·08	0·57*	–0·12	0·01	0·06	0·06	0·57*
	sd	1·03	1·01	0·95	1·01		1·03	1·02	0·94	1·02	
20:4*n*-3	Mean	0·02	0·03	–0·05	0·004	0·96*	0·02	0·03	–0·03	–0·02	0·98*
	sd	1·09	0·92	0·99	1·01		1·09	0·93	0·98	1·01	
20:5*n*-3	Mean	0·67†,‡	–0·67	0·58†,‡	–0·59	< 0·001§	0·56†,‡	–0·56	0·53†,‡	–0·54	< 0·001§
	sd	0·93	0·62	0·77	0·77		0·96	0·74	0·81	0·84	
22:5*n*-3	Mean	–0·01	0·09	–0·14	0·06	0·41*	–0·04	0·08	–0·01	–0·04	0·82*
	sd	1·12	0·99	0·85	1·02		1·03	0·98	0·94	1·06	
22:6*n*-3	Mean	0·60†,‡	–0·66	0·64†,‡	–0·59	< 0·001§	0·38†,‡	–0·43	0·48†,‡	–0·44	< 0·001*
	sd	0·99	0·58	0·92	0·54		0·90	0·91	0·97	0·84	
Total *n*-3 LC-PUFA	Mean	0·64†,‡	–0·67	0·61†,‡	–0·59	< 0·001§	0·40†,‡	–0·43	0·47†,‡	–0·44	< 0·001*
*n*-6/*n*-3 LC-PUFA ratio	Mean						–0·57†,‡	0·65	–0·60†,‡	0·53	< 0·001*
	sd						0·79	0·86	0·72	0·87	

The following variables were natural log-transformed: 18:3*n*-3 %, 20:4*n*-3 %, 20:5*n*-3 %, 18:3*n*-6 μg/ml, 18:3*n*-3 μg/ml, 20:4*n*-3 μg/ml, 20:5*n*-3 μg/ml, 22:5*n*-3 μg/ml, 22:6*n*-3 μg/ml, total *n*-3 PUFA μg/ml.

* One-way ANOVA followed by Tukey’s post hoc test.

§ Welch ANOVA followed by Tamhane’s T’2 post hoc test.

† Significantly different from probiotics *P* < 0·001.

‡ Significantly different from placebo *P* < 0·001.

**Table 3.** *n*-3 long-chain PUFA (LC-PUFA) in serum NEFA as a percentage of total fatty acids (%) and absolute concentration (μg/ml) *z*-scores in pregnant women with overweight and obesity according to the four dietary intervention groups in late pregnancy (Mean values and standard deviations)

		NEFA fatty acids % of total (*z*-scores)					NEFA fatty acids absolute concentration (μg/ml) (*z*-scores)				
		Fish oil + placebo	Probiotics + placebo	Fish oil + probiotics	Placebo + placebo	*P*	Fish oil + placebo	Probiotics + placebo	Fish oil + probiotics	Placebo + placebo	*P*
*n*		90	92	91	88		90	92	91	88	
18:3*n*-3	Mean	–0·07	–0·10	0·01	0·16	0·30*	–0·05	–0·11	0·02	0·15	0·34*
	sd	0·99	0·93	1·07	1·00		1·00	0·91	1·08	0·99	
20:4*n*-3	Mean	0·11	–0·13	0·01	0·02	0·44*	0·11	–0·15	0·01	0·03	0·35*
	sd	0·91	1·06	0·97	1·05		0·91	1·04	0·99	1·05	
20:5*n*-3	Mean	0·27†	–0·25	0·03	–0·05	0·01*	0·28‡	–0·28	0·03	–0·03	0·002*
	sd	0·78	1·04	1·06	1·04		0·83	0·99	1·05	1·05	
22:5*n*-3	Mean	0·01	0·11	–0·08	–0·04	0·63*	0·03	0·07	–0·09	–0·01	0·75*
	sd	0·96	1·01	1·10	0·92		1·00	0·97	1·11	0·92	
22:6*n*-3	Mean	0·33‡,§	–0·29	0·37‡,§	–0·41	< 0·001*	0·31‡,§	–0·31	0·29‡,§	–0·30	< 0·001*
	sd	0·98	0·81	1·07	0·88		1·06	0·81	1·07	0·86	
Total *n*-3 LC-PUFA	Mean	0·31‡,§	–0·32	0·35‡,§	–0·34	< 0·001*	0·28‡,||	–0·34	0·28‡,||	–0·22	< 0·001*
	sd	0·92	0·85	0·93	1·10		1·03	0·83	0·96	1·03	
*n*-6/*n*-3 LC-PUFA ratio	Mean						–0·34‡,§	0·36	–0·37‡,§	0·36	< 0·001*
	sd						0·89	0·73	1·03	1·07	

The following variables were natural log-transformed: 18:3*n*-3 %, 20:4*n*-3 %, 20:5*n*-3 %, 22:5*n*-3 %, 18:3*n*-3 μg/ml, 20:4*n*-3 μg/ml, 20:5*n*-3 μg/ml, 22:5*n*-3 μg/ml, 22:6*n*-3 μg/ml, *n*-3 %, total *n*-3 PUFA μg/ml, *n*-6/*n*-3 PUFA ratio.

* One-way ANOVA followed by Tukey’s post hoc test.

† Significantly different from probiotics *P* < 0·05, *P* < 0·01.

‡ Significantly different from probiotics *P* < 0·001.

|| Significantly different from placebo *P* < 0·01.

§ Significantly different from placebo *P* < 0·001.

**Table 4.** *n*-3 long-chain PUFA (LC-PUFA) in serum cholesteryl esters (CE) as a percentage of total fatty acids (%) and absolute concentration (μg/ml) *z*-scores in pregnant women with overweight and obesity according to the four dietary intervention groups in late pregnancy (Mean values and standard deviations)

		CE fatty acids % of total (*z*-scores)					CE fatty acids absolute concentration (μg/ml) (*z*-scores)				
		Fish oil + placebo	Probiotics + placebo	Fish oil + probiotics	Placebo + placebo	*P*	Fish oil + placebo	Probiotics + placebo	Fish oil + probiotics	Placebo + placebo	*P*
*n*		90	92	91	88		90	92	91	88	
18:3*n*-3	Mean	0·10	–0·06	–0·04	0·01	0·72*	0·10	–0·04	–0·02	–0·04	0·78*
	sd	0·87	0·98	0·95	1·19		0·91	0·98	0·89	1·20	
20:4*n*-3	Mean	0·16	–0·10	–0·01	–0·05	0·36*	0·16	–0·07	0·001	–0·09	0·30*
	sd	0·92	1·01	0·96	1·11		0·90	1·02	1·00	1·07	
20:5*n*-3	Mean	0·54†,‡	–0·46	0·43†,‡	–0·51	< 0·001*	0·46†,‡	–0·38	0·38†,‡	–0·46	< 0·001*
	sd	0·96	0·66	0·94	0·93		1·01	0·73	0·95	0·94	
22:5*n*-3	Mean	–0·10	–0·06	0·10	0·06	0·58*	–0·11	–0·05	0·11	0·04	0·46*
	sd	1·11	1·02	0·95	0·92		1·10	1·01	0·97	0·91	
22:6*n*-3	Mean	0·31†,‡	–0·35	0·29†,‡	–0·25	< 0·001*	0·29†,‡	–0·33	0·30†,‡	–0·27	< 0·001*
	sd	0·84	1·02	0·87	1·08		0·89	1·01	0·86	1·06	
Total *n*-3 LC-PUFA	Mean	0·51†,‡	–0·54	0·42†,‡	–0·38	< 0·001*	0·40†,‡	–0·37	0·25†,‡	–0·29	< 0·001§
	sd	0·93	0·83	0·81	0·98		1·30	0·54	0·99	0·79	
*n*-6/*n*-3 LC-PUFA ratio	Mean						–0·44†,‡	0·52	–0·42†,‡	0·34	< 0·001*
	sd						0·93	0·79	0·94	0·94	

The following variables were natural log-transformed: 18:3*n*-3 %, 20:4*n*-3 %, 20:5*n*-3 %, 22:5*n*-3 %, 22:6*n*-3 %, 18:3*n*-3 μg/ml, 20:4*n*-3 μg/ml, 20:5*n*-3 μg/ml, 22:5*n*-3 μg/ml, 22:6*n*-3 μg/ml, *n*-3 PUFA total %, *n*-6/*n*-3 PUFA ratio.

* One-way ANOVA followed by Tukey’s post hoc test.

§ Welch ANOVA followed by Tamhane’s T’2 post hoc test.

† Significantly different from probiotics *P* < 0·001.

‡ Significantly different from placebo *P* < 0·001.

**Table 5.** *n*-3 long-chain PUFA (LC-PUFA) in serum TAGs as a percentage of total fatty acids (%) and absolute concentration (μg/ml) *z*-scores in pregnant women with overweight and obesity according to the four dietary intervention groups in late pregnancy (Mean values and standard deviations)

		TAG fatty acids % of total (*z*-scores)					TAG fatty acids absolute concentration (μg/ml) (*z*-scores)				
		Fish oil + placebo	Probiotics + placebo	Fish oil + probiotics	Placebo + placebo	*P*	Fish oil + placebo	Probiotics + placebo	Fish oil + probiotics	Placebo + placebo	*P*
*n*		89	92	91	87		89	92	91	87	
18:3*n*-3	Mean	–0·15	0·22	–0·10	0·02	0·07*	–0·10	0·18	–0·11	0·03	0·18*
	sd	1·15	0·65	1·07	1·03		1·17	0·72	1·05	1·00	
20:4*n*-3	Mean	0·14	–0·04	–0·08	–0·01	0·46*	0·15	–0·03	–0·10	–0·02	0·41*
	sd	0·98	0·85	0·97	1·18		1·00	0·88	0·93	1·18	
20:5*n*-3	Mean	0·53†,‡	–0·39	0·37†,‡	–0·51	< 0·001*	0·54†,‡	–0·39	0·34†,‡	–0·48	< 0·001*
	sd	0·91	0·76	0·96	0·93		0·93	0·79	0·94	0·93	
22:5*n*-3	Mean	0·29†,‡	–0·30	0·28†,§	–0·27	< 0·001*	0·19||	–0·16	0·17	–0·21	0·01*
	sd	1·05	0·79	1·01	0·98		1·09	0·87	0·95	1·03	
22:6*n*-3	Mean	0·65†,‡	–0·54	0·57†,‡	–0·69	< 0·001*	0·58†,‡	–0·47	0·48†,‡	–0·59	< 0·001*
	sd	0·82	0·59	0·97	0·74		0·85	0·74	0·97	0·81	
Total *n*-3 LC-PUFA	Mean	0·62†,‡	–0·50	0·49†,‡	–0·61	< 0·001¶	0·52†,‡	–0·43	0·37†,‡	–0·47	< 0·001¶
	sd	0·86	0·62	1·07	0·73		1·29	0·54	1·03	0·46	
*n*-6/n3 LC-PUFA ratio	Mean						–0·64†,‡	0·55	–0·53†,‡	0·62	< 0·001¶
	sd						0·89	0·63	0·90	0·82	

The following variables were natural log-transformed: 18:3*n*-3 %, 20:4*n*-3 %, 20:5*n*-3 %, 22:6*n*-3 %, 20:4*n*-3 μg/ml, 20:5*n*-3 μg/ml, 22:5*n*-3 μg/ml, 22:6*n*-3 μg/ml, *n*-3 PUFA total %, *n*-6/*n*-3 PUFA ratio.

* One-way ANOVA followed by Tukey’s post hoc test.

¶ Welch ANOVA followed by Tamhane’s T’2 post hoc test.

† Significantly different from probiotics *P* < 0·001.

‡ Significantly different from placebo *P* < 0·001.

§ Significantly different from placebo *P* < 0·01.

|| Significantly different from placebo *P* < 0·05.

The dietary intervention had an impact on the total *n*-6 LC-PUFA in serum PC but not in NEFA, CE or TAG. The % of total *n*-6 LC-PUFA in the serum PC was statistically significantly lower in the fish oil and fish oil + probiotics groups compared with the probiotics and placebo groups (*P* ≤ 0·001 for all comparisons). The % of LA (18:2*n*-6) in serum PC was lower in the fish oil + probiotics group compared with the probiotics group (*P* = 0·03). The % of eicosadienoic acid (EDA, 20:2*n*-6) was lower in the fish oil group compared with the probiotics (*P* = 0·001) or placebo groups (*P* = 0·01) and lower in the fish oil + probiotics group compared with the probiotics group (*P* = 0·01). The % of DGLA (20:3*n*-6) was lower in the fish oil and fish oil + probiotics groups compared with the probiotics (*P* < 0·001 in both comparisons) and placebo groups (*P* = 0·002 in both comparisons). The concentrations of EDA (20:2*n*-6) and DGLA (20:3*n*-6) were lower in the fish oil group compared with the probiotics group (*P* = 0·005 and *P* = 0·01, respectively). In serum CE, the % of DGLA (20:3*n*-6) differed between the intervention groups (overall *P* = 0·03), but after Tukey’s post hoc analysis there were no significant effects in pair-wise comparisons between groups. In serum TAG, the % of EDA (20:2*n*-6) was lower in the fish oil group compared with the probiotics (*P* = 0·01) and placebo groups (*P* = 0·002). The finding was similar when expressed as concentration regarding the comparison between fish oil and placebo groups (*P* = 0·03). The results of the effect of the intervention on *n*-6 LC-PUFA are presented in online Supplementary Tables 2–5 (PC, NEFA, CE and TAG, respectively).

The *n*-6/*n*-3 PUFA ratio in serum differed statistically significantly between the intervention groups in all four fractions, being lower in the fish oil and fish oil + probiotics groups compared with the probiotics and placebo groups for all four fractions (*P* < 0·001, Tables 1–4)

The results for other fatty acids (i.e. SFA as well as MUFA) are shown in online Supplementary Tables 6–9. The dietary intervention had a statistically significant effect on some SFA and MUFA in serum PC and TAG and on MUFA in serum NEFA, whereas no effect was observed for either SFA or MUFA in serum CE (see details in the online Supplementary Tables 6–9).

The raw % values of all fatty acids according to the intervention groups are presented in online Supplementary Tables 10–13.

Furthermore, the effect of the intervention on the change from early to late pregnancy of the fatty acids, which differed statistically significantly between the intervention groups in late pregnancy, was investigated (PC, NEFA, CE and TAG in online Supplementary Tables 14–17, respectively). The results remained essentially the same as they were in late pregnancy analyses except for the absolute concentration of eicosenoic acid (20:1*n*-9) in serum NEFA and total fatty acids in % in serum PC which were no longer statistically significant after adjusting for early pregnancy values, and further some pair-wise comparisons related to DGLA (20:3*n*-6) in serum PC and CE become evident and that for EDA (20:2*n*-6) in serum TAG disappeared.

The effect of the intervention on *n*-3 LC-PUFA was additionally evaluated in two-factorial design. In serum PC, CE and TAG, both % and concentration of total *n*-3 LC-PUFA, EPA (20:5*n*-3) and DHA (22:6*n*-3) were higher and ratio of *n*-6 to *n*-3 LC-PUFA was lower in women receiving fish oil compared with women who did not receive fish oil (*P* < 0·001, online Supplementary Tables 18, 20 and 21). In serum NEFA, both % and concentration of total *n*-3 LC-PUFA and EPA (20:5*n*-3) and % of DHA (22:5*n*-3) were higher and ratio of *n*-6 to *n*-3 LC-PUFA was lower in women receiving fish oil compared with women who did not receive fish oil (all *P* < 0·004, online Supplementary Table 19). Interestingly, women receiving probiotics had lower % and concentration of EPA (20:5*n*-3) as compared with women not receiving probiotics (*P* = 0·03 and *P* = 0·02, respectively, online Supplementary Table 15). In serum TAG, % of α-linolenic acid (ALA) (18:3*n*-3) was lower (*P* = 0·02) and % and concentrations of DPA were higher in women receiving fish oil compared with women who did not receive fish oil (*P* < 0·001, online Supplementary Table 21).

### The *n*-3 LC-PUFA of four lipid fractions in relation to low-grade inflammation

**Fig. 2.**
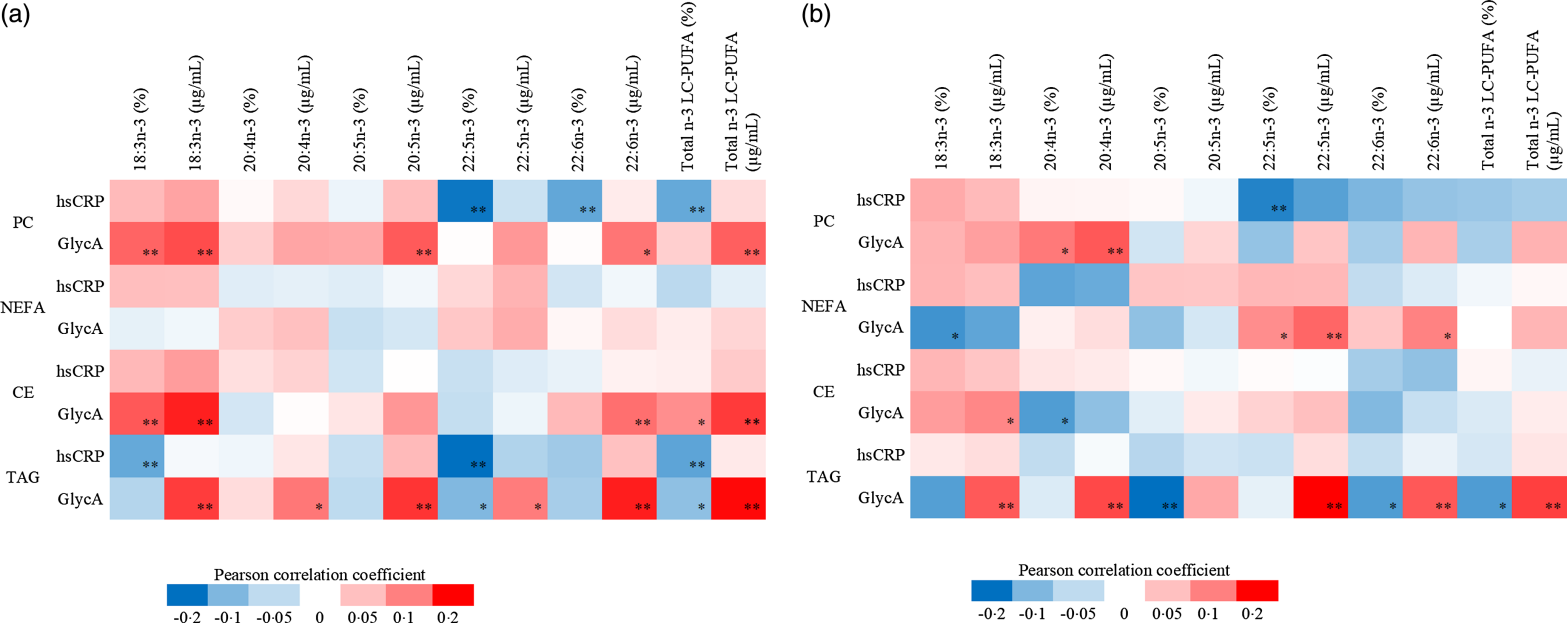
(a) The heatmap describing the Pearson correlation coefficients between early pregnancy serum hsCRP and GlycA and *n*-3 LC-PUFA in PC, NEFA, CE and TAG. Red colour indicates positive correlations while blue negatives, ** *P* < 0·01, * *P* < 0·05. *n* varies between 359 and 361. The following variables were natural log-transformed: hsCRP, PC 18:3*n*-3 %, PC 18:3*n*-3 μg/ml, PC 20:4*n*-3 %, PC 20:4*n*-3 μg/ml, PC 20:5*n*-3 %, PC 20:5*n*-3 μg/ml, PC 22:5*n*-3 μg/ml, NEFA 18:3*n*-3 %, NEFA 18:3*n*-3 μg/ml, NEFA 20:4*n*-3 %, NEFA 20:4*n*-3 μg/ml, NEFA 20:5*n*-3 %, NEFA 20:5*n*-3 μg/ml, NEFA 22:5*n*-3 μg/ml, NEFA 22:6*n*-3 %, NEFA 22:6*n*-3 μg/ml, CE 18:3*n*-3 %, CE 18:3*n*-3 μg/ml, CE 20:4*n*-3 %, CE 20:4*n*-3 μg/ml, CE 20:5*n*-3 %, CE 20:5*n*-3 μg/ml, CE 22:5*n*-3 %, CE 22–5n:3 μg/ml, CE 22:6*n*-3 %, CE 22:6*n*-3 μg/ml, TAG 18:3*n*-3 %, TAG 18:3*n*-3 μg/ml, TAG 20:4*n*-3 %, TAG 20:4*n*-3 μg/ml, TAG 20:5*n*-3 %, TAG 20:5*n*-3 μg/ml, TAG 22:5*n*-3 μg/ml, TAG 22:6*n*-3 %, TAG 22:6*n*-3 μg/ml, PC *n*-3 total μg/ml, NEFA *n*-3 total %, NEFA *n*-3 total μg/ml, CE *n*-3 total %, CE *n*-3 total μg/ml, TAG *n*-3 total %. (b) The heatmap describing the Pearson correlation coefficients between late pregnancy serum hsCRP and GlycA and *n*-3 LC-PUFA in PC, NEFA, CE and TAG. Red colour indicates positive correlations while blue negatives, ** *P* < 0·01, * *P* < 0·05. *n* varies between 307 and 311. The following variables were natural log-transformed: hsCRP, PC 18:3*n*-3 %, PC 18:3*n*-3 µg/ml, PC 20:4*n*-3 %, PC 20:4*n*-3 µg/ml, PC 20:5*n*-3 %, PC 20:5*n*-3 µg/ml, PC 22:5*n*-3 µg/ml, PC 22:6*n*-3 µg/ml, NEFA 18:3*n*-3 %, NEFA 18:3*n*-3 µg/ml, NEFA 20:4*n*-3 %, NEFA 20:4*n*-3, µg/ml, NEFA 20:5*n*-3 %, NEFA 20:5*n*-3 µg/ml, NEFA 22:5*n*-3 %, NEFA 22:5*n*-3 µg/ml, NEFA 22:6*n*-3 %, NEFA 22:6*n*-3 µg/ml, CE 18:3*n*-3 %, CE 18:3*n*-3 µg/ml, CE 20:4*n*-3 %, CE 20:4*n*-3 µg/ml, CE 20:5*n*-3 %, CE 20:5*n*-3 µg/ml, CE 22:5*n*-3 %, CE fatty acid 22:5*n*-3 µg/ml, CE 22:6*n*-3 %, CE 22:6*n*-3 µg/ml, TAG 18:3*n*-3 %, TAG 18:3*n*-3 µg/ml, TAG 20:4*n*-3 %, TAG 20:4*n*-3 µg/ml, TAG 20:5*n*-3 %, TAG 20:5*n*-3, µg/ml, TAG 22:5*n*-3 µg/ml, TAG 22:6*n*-3 %, TAG 22:6*n*-3 µg/ml, PC *n*-3 µg/ml, NEFA *n*-3 total %, CE *n*-3 total %, TAG *n*-3 total %, NEFA *n*-3 total µg/ml. hsCRP, high-sensitivity C-reactive protein; PC, phosphatidylcholine; CE, cholesteryl ester.

Out of all forty-eight *n*-3 LC-PUFA variables evaluated as % and concentration in early and late pregnancy, GlycA correlated both positively and inversely but weakly with 18 *n*-3 LC-PUFA variables in early pregnancy and with 16 in late pregnancy, while hsCRP showed weak inverse correlations with six variables in early pregnancy and one in late pregnancy (Fig. 2(a) and (b)).

In early pregnancy, positive correlations with GlycA were observed for ALA (18:3*n*-3), DHA (22:6*n*-3) and total *n*-3 LC-PUFA in serum PC, CE and TAG and for EPA (20:5*n*-3) in serum PC and TAG (all *r* < 0·300, *P* < 0·01), whereas inverse correlations for DPA (22:5*n*-3) and total *n*-3 LC-PUFA in serum TAG were observed. hsCRP correlated inversely with DPA (22:5*n*-3), DHA (22:6*n*-3) and total *n*-3 LC-PUFA in serum PC, and with ALA (18:3*n*-3), DPA (22:5*n*-3) and total *n*-3 LC-PUFA in serum TAG (all *r* > –0·300, *P* < 0·01, findings either in both units or % or concentration (see details in heat map Fig. 2(a)). Neither GlycA nor hsCRP correlated with fatty acids in serum NEFA in early pregnancy.

In late pregnancy, GlycA correlated positively with eicosatetraenoic acid (ETA, 20:4*n*-3) in serum PC, with DPA (22:5*n*-3) and DHA (22:6*n*-3) in serum NEFA, with ALA (18:3*n*-3) in serum CE and with ALA (18:3*n*-3), ETA (20:4*n*-3) DPA (22:5*n*-3), DHA (22:6*n*-3) and total *n*-3 LC-PUFA in serum TAG while inversely with ALA (18:3*n*-3) in serum NEFA, with ETA (20:4*n*-3) in serum CE and with EPA (20:5*n*-3), DHA (22:6*n*-3) and total *n*-3 LC-PUFA in serum TAG. hsCRP correlated inversely with DPA (22:5*n*-3) in serum PC (all *r* < 0·300 or >–0·300, *P* < 0·01, findings either in both units or % or concentration (see details in heat map Fig. 2(b))).

### The *n*-3 and *n*-6 LC-PUFA of four lipid fractions in relation to the risk of developing gestational diabetes mellitus

**Fig. 3.**
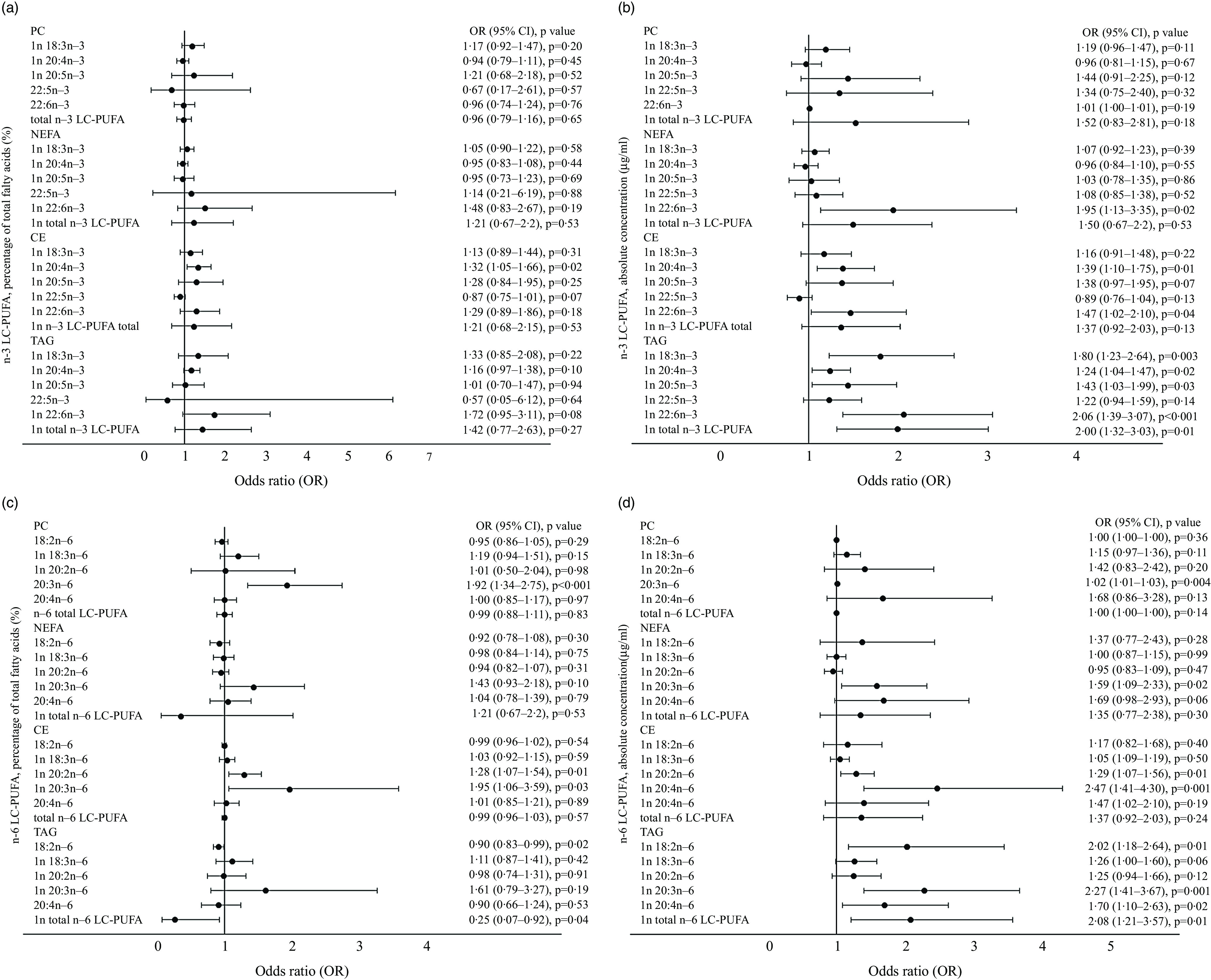
(a)–(d) The association of *n*-3 LC-PUFA in early pregnancy evaluated as proportion of total fatty acids (%) (panel a) and absolute concentration (µg/ml) (panel b) in serum PC, NEFA, CE and TAG and the risk of developing GDM. The association of *n*-6 LC-PUFA in early pregnancy evaluated as proportion of total fatty acids (%) (panel c) and absolute concentration (µg/ml) (panel d) in serum PC, NEFA, CE and TAG and the risk of developing GDM. GDM positive, *n* 81; GDM negative PC *n* 274, NEFA, CE and TAG *n* 275. Logistic regression, adjusted for intervention group, natural log-transformed variables are labelled with ‘ln’. PC, phosphatidylcholine; CE, cholesteryl ester; GDM, gestational diabetes mellitus.

Regarding the association between the *n*-3 LC-PUFA and the risk of developing GDM, ALA (18:3*n*-3), ETA (20:4*n*-3), EPA (20:5*n*-3), DHA (22:6*n*-3) and total *n*-3 LC-PUFA were associated with an increased risk for GDM (Fig. 3(a) and (b)). Specifically, the % and concentration of ETA (20:4*n*-3) in serum CE were associated with 32 % (*P* = 0·02) and 39 % (*P* = 0·01) increased risk of GDM, respectively, and the concentration of DHA (22:6*n*-3) in serum CE was associated with 47 % increased risk of GDM (*P* = 0·04). In serum TAG, the concentrations of ALA (18:3*n*-3), ETA (20:4*n*-3), EPA (20:5*n*-3), DHA (22:6*n*-3) and total *n*-3 LC-PUFA were associated with 80 % (*P* = 0·003), 24 % (*P* = 0·02), 43 % (*P* = 0·03), 106 % (*P* < 0·001) and 100 % (*P* = 0·001) increased risk of GDM, respectively.

Out of all *n*-6 LC-PUFA (Fig. 3(c) and (d)), the % of DGLA (20:3*n*-6) in serum PC and in serum CE was associated with 92 % (*P* < 0·001) and 95 % (*P* = 0·03) increased risk of GDM, respectively. The concentration of DGLA (20:3*n*-6) in serum PC, NEFA, CE and TAG was associated with 2 % (*P* = 0·004), 59 % (*P* = 0·01), 147 % (*P* = 0·001) and 127 % (*P* = 0·001) increased risk for GDM, respectively. Also, % and concentration of EDA (20:2*n*-6) in serum CE as well as concentration of LA (18:2*n*-6), arachidonic acid (AA) (20:4*n*-6) and total *n*-6 LC-PUFA in serum TAG were associated with 29 % (*P* = 0·01), 102 % (*P* = 0·01), 70 % (*P* = 0·01) and 108 % (*P* = 0·01) increased risk of GDM, respectively, while the % of LA (18:2*n*-6) and total *n*-6 LC-PUFA in serum TAG decreased risk of GDM by 10 % (*P* = 0·02) and 75 % (*P* = 0·04), respectively.

The fatty acids related to the onset of GDM in the univariate logistic regression analyses (*P* < 0·05) were analysed in multivariate logistic regression analyses with and without intakes of total fat and SFA (g). Out of fourteen models constructed, % DGLA in serum PC remained significant notwithstanding including the intake of total fat (OR 1·7, 95 %CI 1·1, 2·6, *P* = 0·02 and OR 1·7, 95 %CI 1·1, 2·6, *P* = 0·02) or SFA (OR 1·7, 95 %CI 1·1, 2·6, *P* = 0·02 and OR 1·7 95 %CI 1·1, 2·6, *P* = 0·01) and without the intake of total fat or SFA (OR 1·7, 95 %CI 1·1, 2·5, *P* = 0·02 and OR 1·7, 95 %CI 1·1, 2·5, *P* = 0·02) in two models.

## Discussion

We demonstrate that fish oil and the combination of fish oil and probiotics had an impact on the sFA profile in pregnant women with overweight and obesity whilst against our expectations probiotics did not when the four intervention groups were compared. However, when women receiving probiotics were compared against women not receiving probiotics, EPA in serum NEFA was lower in those who received probiotics as compared with those who did not. Low-grade inflammation was related to serum *n*-3 PUFA, which were further related to the risk of GDM. To our knowledge, no prior studies evaluating the effect of the combination of fish oil and probiotics on sFA exist. Although plausible, and suggested by previous studies^(8–10)^, in this study, the effect of a combination of fish oil and probiotics on sFA composition was not significantly different from that of fish oil alone.

Interestingly, the impact of the intervention on some *n*-3 and *n*-6 LC-PUFA was only evident in the fish oil group but not in the combination group and we suggest that it may be that the probiotics may inhibit the action of fish oil, as seen for EDA in serum PC and TAG: the combination group did not differ from the placebo group as was the case for fish oil supplementation alone. We showed that *n*-3 LC-PUFA levels, including total *n*-3 LC-PUFA, EPA, DHA and DPA, were higher in the fish oil and fish oil + probiotics groups compared with placebo, and *n*-6 LC-PUFA levels, including total *n*-6 LC-PUFA, LA, EDA and DGLA, were lower in the fish oil and/or fish oil + probiotics groups compared with placebo. When the groups were combined in two-way ANOVA analysis, the effect of the fish oil on the *n*-3 LC-PUFA was strengthened when compared with the women not receiving fish oil. Interestingly, AA was not affected by the intervention. The *n*-6/*n*-3 PUFA ratio was lower in the fish oil and fish oil + probiotics groups compared with placebo and the intervention was associated with differences in the concentration and % of some SFA and MUFA. Previous studies evaluating the effect of fish oil on sFA in pregnant women have shown that the percentage of DHA in serum PL increased in pregnant women who were instructed to consume daily fish oil supplements, in a smaller dose than used in our study, 120 mg DHA and 180 mg EPA daily, from week 21 of pregnancy to delivery^(9)^. Similarly, 0·5 g DHA and 0·15 g EPA^(26)^ and 0·2 g DHA^(27)^ have been able to increase DHA in plasma and erythrocyte PL, respectively. In another study, where the amount of DHA and EPA was relatively high, in pregnant women who consumed 1·1 g EPA plus 2·2 g DHA per day from 20 gestational weeks until delivery, EPA and DHA in erythrocyte PL were higher and *n*-6 LC-PUFA lower than in control group in late pregnancy^(28)^. Also, the intake of oily fish is related to increased percentages of total *n*-3 LC-PUFA and DHA in serum PL^(29)^ and percentages of EPA and DHA in plasma PC^(10)^ in pregnant women.

Less is known about the effects of probiotics on circulating fatty acid levels. In a study with non-pregnant adults with hypertriacylglycerolaemia, *L. gasseri* SBT2055 decreased serum NEFA levels^(11)^. In experimental animals, probiotics have been shown to alter liver fatty acid metabolism via interfering with high-fat diet-induced mitochondrial dysfunction^(30)^. However, we were not able to demonstrate an effect of probiotics on fatty acid levels in the four group comparisons, but in the combined probiotics groups an effect on % of EPA in serum NEFA was shown: women who received probiotics had lower % of EPA as compared with women who did not receive probiotics. In one previous study, although with small number of study participants (*n* 25) and differing methods for lipid analysis^(31)^, no changes in fatty acids in serum PC, CE, TAG or other lipids after 3 week *L. rhamnosus* GG intervention in non-pregnant subjects were shown. In our previous study, serum DHA and *n*-3 fatty acids, the ratio of DHA, *n*-3 fatty acids, PUFA and MUFA to total fatty acids as well as other lipid metabolites measured by NMR spectroscopy were changed after fish oil and/or probiotic intervention in pregnant women without GDM^(13)^. We propose that more studies are needed to investigate the effects of probiotics in pregnant women; the use of omics methods might be an asset here.

We demonstrated that sFA are related to risk for GDM: a higher level of *n*-3 LC-PUFA, namely ALA, ETA, EPA and DHA and total *n*-3 LC-PUFA, and *n*-6 LC-PUFA, namely LA, EDA, DGLA and AA and total *n*-6 LC-PUFA, was associated with an increased risk of GDM. Also, two *n*-6 LC-PUFA measurements, % of LA and total *n*-6 LC-PUFA, were associated with a decreased risk of GDM. The fatty acids observed to be related to the onset of GDM were mainly in serum TAG and CE. In serum PC, only DGLA was related to GDM. Previous studies conducted to study fatty acids and GDM are cross-sectional^(32,33)^ as well as prospective^(7,34,35)^ as is our study; we evaluated the relation of fatty acid levels in early pregnancy to the development of GDM in mid-pregnancy when screening is conducted according to the National Current Care Guidelines. The other prospective studies have reported that (a) higher concentrations of ALA and DHA in serum CE at gestational weeks 11–14 are associated with higher risk of GDM later in pregnancy^(35)^; (b) higher percentage of LA and a higher *n*-6/*n*-3 PUFA ratio in erythrocyte total lipids in early pregnancy in women who developed GDM^(7)^ and (c) higher concentration of EPA in women who developed GDM^(34)^ compared with women who did not. These observations are in line with our findings. In contrast to our results, one study showed lower levels of DHA, LA and AA in erythrocyte total lipids in early pregnancy in women who developed GDM^(7)^. It seems that more studies involving women with GDM are needed due to the inconsistent findings and different lipid pools and fractions assessed, which may affect the results and their interpretation. It is of note that the diet may affect the development of GDM as we have reported previously; the dietary intake of total fat, saturated fat and trans fatty acids was related to the development of GDM^(36)^. In this study, the intakes of total fat and SFA did not affect the findings related to DGLA.

One mechanism that may link fatty acids and GDM risk is inflammation-mediated alterations in insulin metabolism. The production of pro-inflammatory eicosanoids via increased levels of precursor AA, which was associated with higher odds for GDM in our study, can increase the levels of some other inflammatory factors, for example, some cytokines, as reviewed by Calder^(37)^. LC-PUFA can also directly affect the activity of transcription factors regulating expression of genes encoding several inflammatory factors^(37)^. In turn, increased levels of inflammatory factors may contribute to insulin resistance by affecting insulin signalling pathways^(38)^. Another mechanism could be that the *n*-6 and *n*-3 LC-PUFA have an effect on deposition of adipose tissue (reviewed in Buckley *et al*.^(39)^ and Muhlhausler *et al*.^(40)^) as discussed also by Vidakovic *et al*.^(41)^ and which further can affect glucose metabolism and low-grade inflammation.

We found that correlations between hsCRP and *n*-3 LC-PUFA were only negative, which is in agreement with the consensus about the anti-inflammatory effects of *n*-3 LC-PUFA. The correlations between GlycA and *n*-3 LC-PUFA were both negative and positive depending on the fatty acid and fraction. We suggest that the relation of GlycA with fatty acids is more complex, since GlycA consists of multiple acute phase proteins^(25)^, which may influence the results. Another reason could be that GlycA is more sensitive at detecting associations, and the positive and negative associations arise from the correlations between GlycA and the intermediate phases of synthesis of fatty acids, that is, processes of elongation and desaturation. We found similar associations between low-grade inflammatory markers and fatty acids using NMR spectroscopy in early pregnancy (*n* 99)^(42)^. And it is of note that in our study, the intervention did not affect serum hsCRP^(43)^.

Plasma or serum contains fatty acids in multiple chemical forms (TAG, CE, PL and NEFA). Here, we report the fatty acid composition of each of these pools. TAG are carried mainly in chylomicrons (of gut origin in the fed state) and VLDL (of hepatic origin in the fasting state) and are a transport pool of fatty acids being delivered to peripheral tissues. CE are mainly carried in VLDL remnants (ultimately LDL) and are a transport pool of fatty acids being delivered to peripheral tissues and to the liver. PL are found in the monolayer that coats all lipoproteins ensuring their solubility in the aqueous bloodstream; the main PL in human plasma and serum is PC. The fatty acid composition of circulating PL, including PC, is related to the fatty acid composition of many cell types. In the fasting state, NEFA are mainly fatty acids released from adipose tissue lipolysis and so their composition represents that of adipose tissue TAG. Thus, the fatty acids in different plasma lipid pools align with different metabolic or functional roles. It is well described that the fatty acid composition of each of these pools is modified by an increased intake of EPA and DHA^(44–48)^. Currently, there is no strong consensus on whether to use whole plasma or serum or isolated lipid pools in trials involving fatty acids^(49)^ and the choice of fraction also depends on which fatty acid and pool is of most interest^(50)^. According to our study, serum PC demonstrated to be the most useful in indicating the response of the intervention in the *n*-6 and *n*-3 LC-PUFA.

We report fatty acids in each pool as both weight percentage and absolute concentration. Weight % describes the concentration of each fatty acid relative to all fatty acids in the pool. It is the most common way of reporting fatty acid composition data^(49)^, has particular advantages and allows easy comparison with much of the existing literature. One advantage of using weight % is that fatty acids of different families (e.g. *n*-6 and *n*-3) often have opposing actions and so a comparison of the relative contribution of each individual fatty acid or an entire family of fatty acids (i.e. as weight %) provides an idea of the degree of dominance of one over another. However, weight % ignores the actual size of the pool in which the fatty acid is found. Thus, a fatty acid with a higher % in a small pool may have a lower absolute concentration than a fatty acid with a low % in a large pool and vice versa. Thus, reporting fatty acids as absolute concentrations provides an idea of the true exposure of cells and tissues to different fatty acids within a particular lipid pool. Again, Brenna *et al*.^(49)^ discuss the merits of reporting fatty acids as weight % and as absolute concentration but make no recommendation of which should be used, saying both are meaningful ways of reporting fatty acid concentrations.

One strength of our study is its randomised placebo-controlled design, which is the gold standard design for studies evaluating the impact of dietary interventions on any clinical or laboratory measure. Another strength is the high number of participants who gave a blood sample after at least 9 h over night fasting for the analysis of the fatty acids. Even though the power calculations were done based on the reduction of incidence of GDM and fasting glucose levels^(14)^, we are confident that the power with this high number of study subjects is sufficient to detect changes in fatty acid levels after the dietary intervention, compared with previous reports with, for example, *n* 40^(28)^, *n* 48^(27)^, *n* 61–64^(26)^ and *n* 67–68^(9)^ pregnant women per intervention group. Moreover, we analysed all four lipid fractions and expressed fatty acids in two units, concentration and percentage of total fatty acids; percentage is more commonly used in research as it requires less resources. However, by measuring only the percentages, one may miss findings related to the differences in group-wise comparisons as well as time-wise if all the fatty acids of interest change in the same manner. The contradictory finding related to associations of LA and total *n*-6 LC-PUFA with both decreased and increased risk of GDM may be explained by this. One limitation of the current study is the lack of normal weight pregnant women, as a comparison group, as the effect of *n*-3 LC-PUFA supplementation on fatty acid levels may differ according to BMI^(51)^ and the levels may be altered in obesity^(34,41)^ meaning that the generalisation of the results to normal weight pregnant women is limited. However, currently the prevalence of overweight and obesity (41·9 % and 17·0% of Finnish parturients, respectively^(52)^) in pregnancy is increasing, making this a common group in well-woman clinics, and those women are at increased risk for development of metabolic diseases, the reason we chose to study this group of pregnant women. Indeed, new means for preventing disease are in demand, and targeting at higher risk group is reasonable. Obesity may also alter the synthesis of anti-inflammatory oxylipins derived from *n*-3 LC-PUFA in white adipose tissue as shown in a recent study in non-pregnant individuals^(53)^; this could contribute to the inflammation resolving effects of *n*-3 LC-PUFA in obesity.

### Conclusions

We showed that fish oil administered from early pregnancy onwards increased the *n*-3 LC-PUFA levels in the serum of pregnant women with overweight and obesity. As *n*-3 LC-PUFA levels tend to decrease during pregnancy, as we showed in our study, *n*-3 LC-PUFA supplementation during pregnancy may allow an increase in pregnant women’s *n*-3 LC-PUFA status and further greater provision for the fetus. Probiotics showed no impact in the comparisons of the four intervention groups, nor was there an added benefit of combining probiotics with fish oil. Interestingly, EPA in serum NEFA was lower in women receiving probiotics compared with those not receiving probiotics. Decreased low-grade inflammation, measured with the traditional marker hsCRP, was linked with increased *n*-3 LC-PUFA levels, which were further linked with GDM. In contrast, GlycA, a novel low-grade inflammatory marker, showed distinct associations, suggesting that more studies on GlycA and its relation to fatty acids are needed and might provide further insights.

## Supplementary material

For supplementary material accompanying this paper visit https://doi.org/10.1017/S0007114523001915. click here to view supplementary material
