# Supplementary material for: The impact of fish oil and/or probiotics on serum fatty acids and the interaction with low-grade inflammation in pregnant women with overweight and obesity: secondary analysis of a randomised controlled trial
Source: Br J Nutr. 2023 Aug 29;131(2):296–311. doi: 10.1017/S0007114523001915 (PMC10751948; doi:10.1017/S0007114523001915)
Supplement: Supplementary file 1 [file S0007114523001915sup001.docx]

Supplementary Table 1. The n-3 and n-6 long-chain polyunsaturated fatty acids (LC-PUFAs) and their total amounts in phosphatidylcholine (PC), non-esterified fatty acids (NEFAs), cholesterol esters (CEs) and triacylglycerol (TAGs) as % of total fatty acids and absolute concentration (μg/mL) z-scores change on from early to late pregnancy in placebo group. Data are expressed as mean and SD and 95% CI for mean change.

|  |  | Phosphatidylcholine (PC) | | Non-esterified fatty acids (NEFAs) | | Cholesterol esters (CEs) | | Triacylglycerol (TAGs) | |
| --- | --- | --- | --- | --- | --- | --- | --- | --- | --- |
|  |  | **% of total fatty acids** | **Absolute concentration (μg/mL)** | **% of total fatty acids** | **Absolute concentration (μg/mL)** | **% of total fatty acids** | **Absolute concentration (μg/mL)** | **% of total fatty acids** | **Absolute concentration (μg/mL)** |
| 18:3n-3 | n | 85 | 85 | 85 | 85 | 85 | 85 | 85 | 85 |
|  | mean change | 0.12 | 0.11 | 0.16 | 0.14 | 0.15 | 0.13 | -0.09 | 0.01 |
|  | SD | 1.08 | 1.08 | 0.99 | 0.99 | 1.09 | 1.25 | 0.97 | 1.03 |
|  | 95% CI upper bound | -0.12 | -0.12 | -0.06 | -0.07 | -0.08 | -0.14 | -0.29 | -0.22 |
|  | 95% CI lower bound | 0.35 | 0.34 | 0.37 | 0.36 | 0.39 | 0.40 | 0.12 | 0.23 |
| 20:4n-3 | n | 86 | 86 | 86 | 86 | 86 | 86 | 85 | 85 |
|  | mean change | -0.12 | -0.10 | 0.02 | 0.01 | -0.06 | -0.07 | -0.03 | -0.01 |
|  | SD | 1.08 | 1.06 | 0.95 | 0.93 | 0.92 | 0.94 | 1.21 | 1.20 |
|  | 95% CI upper bound | -0.35 | -0.32 | -0.19 | -0.19 | -0.26 | -0.27 | -0.29 | -0.27 |
|  | 95% CI lower bound | 0.11 | 0.13 | 0.22 | 0.21 | 0.14 | 0.13 | 0.23 | 0.25 |
| 20:5n-3 | n | 85 | 85 | 85 | 85 | 85 | 85 | 85 | 85 |
|  | mean change | -0.64 | -0.62 | -0.07 | -0.07 | -0.55 | -0.55 | -0.56 | -0.52 |
|  | SD | 0.73 | 0.78 | 0.99 | 1.05 | 0.82 | 0.85 | 0.88 | 0.90 |
|  | 95% CI upper bound | -0.79 | -0.79 | -0.28 | -0.30 | -0.73 | -0.74 | -0.75 | -0.71 |
|  | 95% CI lower bound | -0.48 | -0.45 | 0.14 | 0.15 | -0.37 | -0.37 | -0.36 | -0.32 |
| 22:5n-3 | n | 86 | 86 | 86 | 86 | 86 | 86 | 85 | 85 |
|  | mean change | 0.03 | -0.01 | -0.06 | -0.07 | -0.05 | -0.05 | -0.12 | -0.13 |
|  | SD | 0.92 | 0.88 | 0.95 | 0.99 | 0.90 | 0.93 | 0.87 | 0.96 |
|  | 95% CI upper bound | -0.16 | -0.20 | -0.27 | -0.28 | -0.25 | -0.25 | -0.31 | -0.34 |
|  | 95% CI lower bound | 0.23 | 0.18 | 0.14 | 0.15 | 0.14 | 0.14 | 0.07 | 0.08 |
| 22:6n-3 | n | 85 | 85 | 85 | 85 | 85 | 85 | 85 | 85 |
|  | mean change | -0.68 | -0.55 | -0.50 | -0.40 | -0.16 | -0.22 | -0.73 | -0.60 |
|  | SD | 0.53 | 0.74 | 0.91 | 1.03 | 1.09 | 1.17 | 0.60 | 0.70 |
|  | 95% CI upper bound | -0.79 | -0.71 | -0.69 | -0.62 | -0.39 | -0.47 | -0.86 | -0.75 |
|  | 95% CI lower bound | -0.57 | -0.39 | -0.30 | -0.17 | 0.08 | 0.04 | -0.60 | -0.45 |
| Total n-3 LC-PUFAs | n | 86 | 86 | 86 | 86 | 86 | 86 | 85 | 85 |
|  | mean change | -0.67 | -0.56 | -0.39 | -0.33 | -0.45 | -0.45 | -0.65 | -0.51 |
|  | SD | 0.50 | 0.73 | 1.12 | 1.13 | 0.72 | 0.90 | 0.63 | 0.71 |
|  | 95% CI upper bound | -0.78 | -0.72 | -0.63 | -0.57 | -0.60 | -0.64 | -0.79 | -0.66 |
|  | 95% CI lower bound | -0.56 | -0.40 | -0.15 | -0.09 | -0.29 | -0.26 | -0.52 | -0.36 |
| 18:2n-6 | n | 85 | 85 | 85 | 85 | 85 | 85 | 85 | 85 |
|  | mean change | 0.04 | -0.02 | -0.15 | -0.06 | -0.17 | -0.12 | -0.25 | 0.04 |
|  | SD | 0.85 | 0.85 | 0.92 | 0.98 | 1.04 | 1.25 | 1.07 | 0.90 |
|  | 95% CI upper bound | -0.14 | -0.21 | -0.35 | -0.27 | -0.40 | -0.39 | -0.49 | -0.15 |
|  | 95% CI lower bound | 0.22 | 0.16 | 0.05 | 0.16 | 0.05 | 0.15 | -0.02 | 0.24 |
| 18:3n-6 | n | 85 | 85 | 85 | 85 | 85 | 85 | 85 | 85 |
|  | mean change | -0.04 | -0.06 | -0.04 | -0.04 | 0.00 | -0.02 | -0.14 | -0.08 |
|  | SD | 0.82 | 0.77 | 1.05 | 1.05 | 1.16 | 1.20 | 0.92 | 0.95 |
|  | 95% CI upper bound | -0.22 | -0.23 | -0.26 | -0.27 | -0.25 | -0.28 | -0.34 | -0.29 |
|  | 95% CI lower bound | 0.13 | 0.10 | 0.19 | 0.18 | 0.25 | 0.24 | 0.06 | 0.12 |
| 20:2n-6 | n | 86 | 86 | 86 | 86 | 86 | 86 | 85 | 85 |
|  | mean change | 0.07 | 0.06 | 0.19 | 0.19 | 0.11 | 0.09 | 0.05 | 0.12 |
|  | SD | 0.89 | 0.86 | 1.02 | 1.00 | 1.09 | 1.10 | 1.00 | 0.94 |
|  | 95% CI upper bound | -0.12 | -0.13 | -0.03 | -0.02 | -0.13 | -0.14 | -0.17 | -0.08 |
|  | 95% CI lower bound | 0.27 | 0.24 | 0.41 | 0.41 | 0.34 | 0.33 | 0.26 | 0.32 |
| 20:3n-6 | n | 85 | 85 | 85 | 85 | 85 | 85 | 85 | 85 |
|  | mean change | 0.32 | 0.15 | 0.00 | 0.01 | 0.19 | 0.03 | 0.13 | 0.19 |
|  | SD | 0.88 | 0.87 | 1.02 | 1.10 | 1.14 | 0.94 | 1.01 | 0.95 |
|  | 95% CI upper bound | 0.13 | -0.04 | -0.21 | -0.23 | -0.06 | -0.17 | -0.09 | -0.02 |
|  | 95% CI lower bound | 0.51 | 0.34 | 0.22 | 0.25 | 0.43 | 0.24 | 0.34 | 0.39 |
| 20:4n-6 | n | 85 | 85 | 85 | 85 | 85 | 85 | 85 | 85 |
|  | mean change | 0.11 | 0.04 | 0.01 | -0.05 | 0.01 | -0.13 | -0.12 | -0.01 |
|  | SD | 0.86 | 0.84 | 0.88 | 1.06 | 0.99 | 1.22 | 1.25 | 1.34 |
|  | 95% CI upper bound | -0.07 | -0.14 | -0.18 | -0.28 | -0.20 | -0.39 | -0.39 | -0.30 |
|  | 95% CI lower bound | 0.30 | 0.22 | 0.20 | 0.18 | 0.23 | 0.13 | 0.14 | 0.28 |
| Total n-6 LC-PUFAs | n | 86 | 86 | 86 | 86 | 86 | 86 | 85 | 85 |
|  | mean change | 0.19 | 0.03 | 0.05 | 0.04 | -0.15 | -0.15 | -0.25 | 0.06 |
|  | SD | 0.74 | 0.81 | 0.84 | 0.87 | 0.96 | 1.21 | 1.05 | 0.93 |
|  | 95% CI upper bound | 0.04 | -0.15 | -0.13 | -0.15 | -0.35 | -0.41 | -0.48 | -0.14 |
|  | 95% CI lower bound | 0.35 | 0.20 | 0.23 | 0.22 | 0.06 | 0.11 | -0.03 | 0.26 |

The following variables were natural log-transformed: PC: 18:3n-6 %, 18:3-3 %, 20:2n-6 %, 20:4n-3 %, 20:5n-3 %, 18:3n-6 μg/mL, 18:3n-3 μg/mL, 20:2n-6 μg/mL, 20:4n-6 μg/mL, 20:4n-3 μg/mL, 20:5n-3 μg/mL, 22:5n-3 μg/mL, 22:6n-3 μg/mL, n-3 total, NEFA: 18:3n-6 %, 18:3n-3, 20:2n-6 %, 20:3n-6 %, 20:4n-3 %, 20:5n-3 %, 22:5n-3 %, 22:6n-3 %, 18:2n-6 μg/mL, 18:3n-6 μg/mL, 18:3n-3 μg/mL, 20:2n-6 μg/mL, 20:3n-6 μg/mL, 20:4n-6 μg/mL, 20:4n-3 μg/mL, 20:5n-3 μg/mL, 22:5n-3 μg/mL, 22:6n-3 μg/mL, n-3 total %, n-3 total μg/mL, n-6 total %, n-6 total μg/mL, CE 18:3n-6 %, 18:3n-3 %, 20:2n-6 %, 20:3n-6 %, 20:4n-3 %, 20:5n-3 %, 22:5n-3 %, 22:6n-3 %, 18:2n-6 μg/mL, 18:3n-6 μg/mL, 18:3n-3 μg/mL, 20:2n-6 μg/mL, 20:3n-6 μg/mL, 20:4n-6 μg/mL, 20:4n-3 μg/mL, 20:5n-3 μg/mL, 22:5n-3 μg/mL, 22:6n-3 μg/mL, n-3 total %, n-6 total μg/mL, TAG: 18:2n-6 %, 18:3n-6 %, 18:3n-3 %, 20:2n-6 %, 20:3n-6 %, 20:4n-6 %, 20:4n-3 %, 20:5n.3 %, 22:5n-3 %, 22:6n.3 %, 18:2n-6 μg/mL, 18:3n-6 μg/mL, 18:3n-3 μg/mL, 20:2n-6 μg/mL, 20:3n-6 μg/mL, 20:4n-6 μg/mL, 20:4n-3 μg/mL, 20:5n-3 μg/mL, 22:5n-3 μg/mL, 22:6n-3 μg/mL, n-3 total %, n-6 total %, n-6 total μg/mL

Supplementary Table 2. n-6 long-chain polyunsaturated fatty acids (LC-PUFAs) in serum phosphatidylcholine (PC) as a percentage of total fatty acids (%) and absolute concentration (μg/mL) z-scores in pregnant women with overweight and obesity according to the four dietary intervention groups in late pregnancy. Data are expressed as mean and SD.

|  |  | PC fatty acids % of total (z-scores) | | | | | PC fatty acids absolute concentration (μg/mL) (z-scores) | | | | |
| --- | --- | --- | --- | --- | --- | --- | --- | --- | --- | --- | --- |
|  |  | **Fish oil + placebo** | **Probiotics + placebo** | **Fish oil + probiotics** | **Placebo + placebo** | **P value** | **Fish oil + placebo** | **Probiotics + placebo** | **Fish oil + probiotics** | **Placebo + placebo** | **P value** |
| n |  | 90 | 91 | 91 | 88 |  | 90 | 91 | 91 | 88 |  |
|  | SD | 1.01 | 0.58 | 0.86 | 0.57 |  | 0.92 | 0.90 | 0.93 | 0.86 |  |
| 18:2n-6 | mean | -0.07 | 0.19 | -0.22^\|\|^ | 0.11 | 0.03^*^ | -0.09 | 0.12 | 0.01 | -0.05 | 0.51^*^ |
|  | SD | 0.93 | 1.15 | 0.81 | 1.05 |  | 0.86 | 1.13 | 1.00 | 1.00 |  |
| 18:3n-6 | mean | -0.05 | 0.14 | -0.05 | -0.04 | 0.50^*^ | -0.06 | 0.13 | -0.02 | -0.06 | 0.51^*^ |
|  | SD | 0.98 | 0.92 | 1.03 | 1.07 |  | 0.98 | 0.91 | 1.05 | 1.06 |  |
| 20:2n-6 | mean | -0.28^¶**^ | 0.27 | -0.18^\|\|^ | 0.19 | <0.001^*^ | -0.24^¶^ | 0.25 | -0.06 | 0.05 | 0.01^§^ |
|  | SD | 0.83 | 1.06 | 0.86 | 1.12 |  | 0.78 | 1.18 | 0.95 | 1.00 |  |
| 20:3n-6 | mean | -0.29^†**^ | 0.36 | -0.30^†**^ | 0.23 | <0.001^*^ | -0.21^\|\|^ | 0.25 | -0.08 | 0.04 | 0.02^§^ |
|  | SD | 0.93 | 1.11 | 0.95 | 0.82 |  | 0.80 | 1.14 | 1.06 | 0.92 |  |
| 20:4n-6 | mean | -0.19 | 0.13 | -0.08 | 0.14 | 0.07^*^ | -0.14 | 0.09 | 0.06 | -0.02 | 0.41^*^ |
|  | SD | 0.91 | 0.92 | 1.04 | 1.10 |  | 0.86 | 0.98 | 1.18 | 0.95 |  |
| Total n-6 LC-PUFAs | mean | -0.28^†‡^ | 0.38 | -0.36^†‡^ | 0.26 | <0.001^*^ | -0.13 | 0.15 | 0.01 | -0.04 | 0.31^*^ |
|  | SD | 0.89 | 1.13 | 0.85 | 0.90 |  | 0.84 | 1.10 | 1.07 | 0.96 |  |

^*^One-way ANOVA followed by Tukey’s post-hoc test or ^§^Welch ANOVA followed by Tamhanes T’2 post-hoc test

significantly different from probiotics p<0.05^||^, p<0.01^¶^, p<0.001^†^

significantly different from placebo p<0.01^**^, p<0.001^‡^

The following variables were natural log-transformed: 18:3n-6 %

Supplementary Table 3. n-6 long-chain polyunsaturated fatty acids (LC-PUFAs) in serum non-esterified fatty acids (NEFAs) as a percentage of total fatty acids (%) and absolute concentration (μg/mL) z-scores in pregnant women with overweight and obesity according to the four dietary intervention groups in late pregnancy. Data are expressed as mean and SD.

|  |  | NEFA fatty acids % of total (z-scores) | | | | | NEFA fatty acids absolute concentration (μg/mL) (z-scores) | | | | |
| --- | --- | --- | --- | --- | --- | --- | --- | --- | --- | --- | --- |
|  |  | **Fish oil + placebo** | **Probiotics + placebo** | **Fish oil + probiotics** | **Placebo + placebo** | **P value** | **Fish oil + placebo** | **Probiotics + placebo** | **Fish oil + probiotics** | **Placebo + placebo** | **P value** |
| n |  | 90 | 92 | 91 | 88 |  | 90 | 92 | 91 | 88 |  |
| 18:2n-6 | mean | 0.05 | 0.05 | -0.03 | -0.08 | 0.76^*^ | 0.05 | -0.07 | -0.04 | 0.07 | 0.74^*^ |
|  | SD | 0.92 | 1.07 | 0.91 | 1.10 |  | 1.10 | 0.94 | 1.04 | 0.91 |  |
| 18:3n-6 | mean | 0.12 | -0.03 | -0.15 | 0.06 | 0.28^¶^ | 0.13 | -0.03 | -0.15 | 0.05 | 0.28^¶^ |
|  | SD | 0.93 | 1.01 | 1.01 | 1.04 |  | 0.94 | 1.00 | 1.02 | 1.03 |  |
| 20:2n-6 | mean | 0.01 | -0.05 | -0.06 | 0.11 | 0.63^*^ | 0.00 | -0.07 | -0.05 | 0.13 | 0.53^*^ |
|  | SD | 0.97 | 0.97 | 0.97 | 1.09 |  | 0.97 | 0.95 | 0.99 | 1.09 |  |
| 20:3n-6 | mean | -0.09 | 0.09 | -0.10 | 0.10 | 0.35^*^ | -0.06 | 0.03 | -0.11 | 0.14 | 0.34^*^ |
|  | SD | 1.01 | 0.74 | 1.16 | 1.04 |  | 1.09 | 0.79 | 1.15 | 0.93 |  |
| 20:4n-6 | mean | -0.14 | 0.21 | -0.08 | 0.001 | 0.10^*^ | -0.07 | 0.11 | -0.08 | 0.05 | 0.51^*^ |
|  | SD | 0.93 | 1.03 | 1.02 | 1.00 |  | 1.13 | 0.69 | 1.00 | 1.13 |  |
| Total n-6 LC-PUFAs | mean | -0.10 | 0.11 | -0.07 | 0.06 | 0.42^*^ | -0.02 | -0.04 | -0.06 | 0.12 | 0.61^*^ |
|  | SD | 0.84 | 1.21 | 1.02 | 0.87 |  | 1.07 | 0.95 | 1.11 | 0.83 |  |

^*^One-way ANOVA followed by Tukey’s post-hoc test or ^¶^Welch ANOVA followed by Tamhanes T’2 post-hoc test

significantly different from probiotics p<0.05, p<0.01^†^, p<0.001^‡^

significantly different from placebo p<0.01^||^, p<0.001^§^

The following variables were natural log-transformed: 18:3n-6 %, 20:2n-6 %, 20:3n-6 %, 20:4n-6 %, 22:5n-6 %, 18:3n-6 μg/mL, 20:2n-6 μg/mL, 20:3n-6 μg/mL, 20:4n-6 μg/mL, total n-6 PUFA %, total n-6 PUFA μg/mL

Supplementary Table 4. n-6 long-chain polyunsaturated fatty acids (LC-PUFAs) in serum cholesteryl esters (CEs) as a percentage of total fatty acids (%) and absolute concentration (μg/mL) z-scores in pregnant women with overweight and obesity according to the four dietary intervention groups in late pregnancy. Data are expressed as mean and SD.

|  |  | | CE fatty acids % of total (z-scores) | | | | | CE fatty acids absolute concentration (μg/mL) (z-scores) | | | | |
| --- | --- | --- | --- | --- | --- | --- | --- | --- | --- | --- | --- | --- |
|  |  | **Fish oil + placebo** | | **Probiotics + placebo** | **Fish oil + probiotics** | **Placebo + placebo** | **P value** | **Fish oil + placebo** | **Probiotics + placebo** | **Fish oil + probiotics** | **Placebo + placebo** | **P value** |
| n |  | 90 | | 92 | 91 | 88 |  | 90 | 92 | 91 | 88 |  |
| 18:2n-6 | mean | 0.05 | | 0.07 | -0.06 | -0.06 | 0.72^*^ | 0.03 | 0.04 | -0.003 | -0.07 | 0.88^*^ |
|  | SD | 0.92 | | 0.90 | 1.15 | 1.02 |  | 1.01 | 0.87 | 1.22 | 0.88 |  |
| 18:3n-6 | mean | 0.00 | | 0.03 | -0.07 | 0.05 | 0.87^*^ | -0.01 | 0.04 | -0.05 | 0.02 | 0.94^*^ |
|  | SD | 0.95 | | 1.03 | 0.94 | 1.09 |  | 0.93 | 1.03 | 0.96 | 1.09 |  |
| 20:2n-6 | mean | -0.05 | | 0.09 | -0.06 | 0.02 | 0.72^*^ | -0.05 | 0.10 | -0.03 | -0.02 | 0.74^*^ |
|  | SD | 1.04 | | 0.89 | 1.01 | 1.07 |  | 1.07 | 0.91 | 1.01 | 1.02 |  |
| 20:3n-6 | mean | -0.11 | | 0.18 | -0.19 | 0.13 | 0.03^*^ | -0.07 | 0.17 | -0.09 | -0.01 | 0.29^*^ |
|  | SD | 0.96 | | 1.05 | 0.87 | 1.08 |  | 0.99 | 1.07 | 1.03 | 0.89 |  |
| 20:4n-6 | mean | -0.02 | | 0.03 | -0.03 | 0.02 | 0.98^*^ | 0.02 | 0.02 | 0.03 | -0.07 | 0.88^*^ |
|  | SD | 0.89 | | 1.00 | 1.03 | 1.09 |  | 0.97 | 0.96 | 1.00 | 1.08 |  |
| Total n-6 LC-PUFAs | mean | 0.04 | | 0.09 | -0.09 | -0.04 | 0.59^*^ | 0.03 | 0.06 | 0.001 | -0.09 | 0.75^*^ |
|  | SD | 0.85 | | 0.86 | 1.26 | 0.98 |  | 0.97 | 0.82 | 1.20 | 0.99 |  |

^*^One-way ANOVA followed by Tukey’s post-hoc test or ^§^Welch ANOVA followed by Tamhanes T’2 post-hoc test

significantly different from probiotics p<0.001^†^

significantly different from placebo p<0.001^‡^

The following variables were natural log-transformed: 18:3n-6 %, 20:2n-6 %, 18:2n-6 μg/mL, 18:3n-6 μg/mL, 20:2n-6 μg/mL, 20:3n-6 μg/mL, 20:4n-6 μg/mL, n-6 PUFAs total μg/mL

Supplementary Table 5. n-6 long-chain polyunsaturated fatty acids (LC-PUFAs) in serum triacylglycerols (TAGs) as a percentage of total fatty acids (%) and absolute concentration (μg/mL) z-scores in pregnant women with overweight and obesity according to the four dietary intervention groups in late pregnancy. Data are expressed as mean and SD.

|  |  | TAG fatty acids % of total (z-scores) | | | | | TAG fatty acids absolute concentration (μg/mL) (z-scores) | | | | |
| --- | --- | --- | --- | --- | --- | --- | --- | --- | --- | --- | --- |
|  |  | **Fish oil + placebo** | **Probiotics + placebo** | **Fish oil + probiotics** | **Placebo + placebo** | **P value** | **Fish oil + placebo** | **Probiotics + placebo** | **Fish oil + probiotics** | **Placebo + placebo** | **P value** |
| n |  | 89 | 92 | 91 | 87 |  | 89 | 92 | 91 | 87 |  |
| 18:2n-6 | mean | 0.05 | -0.05 | 0.17 | -0.18 | 0.12^*^ | 0.06 | -0.04 | 0.04 | -0.07 | 0.80^*^ |
|  | SD | 0.83 | 1.29 | 0.89 | 0.89 |  | 0.96 | 1.11 | 1.05 | 0.87 |  |
| 18:3n-6 | mean | -0.07 | 0.13 | -0.07 | 0.01 | 0.46^*^ | -0.05 | 0.11 | -0.06 | 0.004 | 0.64^*^ |
|  | SD | 1.15 | 0.80 | 0.93 | 1.10 |  | 1.14 | 0.83 | 0.92 | 1.09 |  |
| 20:2n-6 | mean | -0.32^**§^ | 0.14 | -0.04 | 0.22 | 0.001^*^ | -0.23^\|\|^ | 0.11 | -0.06 | 0.19 | 0.02^*^ |
|  | SD | 1.08 | 0.85 | 1.07 | 0.91 |  | 1.09 | 0.90 | 1.05 | 0.92 |  |
| 20:3n-6 | mean | -0.18 | 0.20 | -0.03 | 0.01 | 0.08^*^ | -0.10 | 0.16 | -0.10 | 0.03 | 0.24^*^ |
|  | SD | 0.90 | 0.92 | 1.18 | 0.95 |  | 1.08 | 0.86 | 1.11 | 0.92 |  |
| 20:4n-6 | mean | 0.05 | 0.10 | -0.05 | -0.11 | 0.49^*^ | 0.03 | 0.04 | -0.01 | -0.07 | 0.88^*^ |
|  | SD | 0.61 | 0.62 | 1.34 | 1.21 |  | 1.03 | 1.02 | 1.01 | 0.94 |  |
| Total n-6 LC-PUFAs | mean | -0.47 | 0.52 | -0.02 | -0.05 | 0.17^*^ | -0.28 | 0.39 | -0.19 | 0.07 | 0.86^*^ |
|  | SD | 2.72 | 2.67 | 2.79 | 2.70 |  | 4.28 | 3.90 | 4.02 | 3.61 |  |

^*^One-way ANOVA followed by Tukey’s post-hoc test or ^¶^Welch ANOVA followed by Tamhanes T’2 post-hoc test

significantly different from probiotics p<0.05^**^, p<0.001^†^

significantly different from placebo p<0.05^||^, p<0.01^§^, p<0.001^‡^

The following variables were natural log-transformed: 18:2n-6 %, 18:3n-6 %, 20:2n-6 %, 20:4n-6 %, 18:2n-6 μg/mL, 18:3n-6 μg/mL, 20:2n-6 μg/mL, 20:3n-6 μg/mL, n-6 total %, n-6 PUFAs total μg/mL

Supplementary Table 6. Fatty acids in serum phosphatidylcholine (PC) as % of total fatty acids and absolute concentration (μg/mL) z-scores in pregnant women with overweight and obesity according to the four dietary intervention groups in late pregnancy. Data are expressed as mean and SD.

|  |  | PC fatty acids % of total (z-scores) | | | | | PC fatty acids absolute concentration (μg/mL) (z-scores) | | | | |
| --- | --- | --- | --- | --- | --- | --- | --- | --- | --- | --- | --- |
|  |  | **Fish oil + placebo** | **Probiotics + placebo** | **Fish oil + probiotics** | **Placebo + placebo** | **P value** | **Fish oil + placebo** | **Probiotics + placebo** | **Fish oil + probiotics** | **Placebo + placebo** | **P value** |
| n | | 90 | 91 | 91 | 88 |  | 90 | 91 | 91 | 88 |  |
| 14:0 | mean | -0.02 | 0.01 | -0.11 | 0.13 | 0.47^*^ | -0.03 | 0.04 | -0.01 | 0.004 | 0.97^*^ |
|  | SD | 1.01 | 1.00 | 0.94 | 1.06 |  | 0.95 | 1.05 | 0.93 | 1.08 |  |
| 16:0 | mean | 0.01 | -0.06 | -0.02 | 0.08 | 0.81^*^ | -0.05 | 0.04 | 0.11 | -0.09 | 0.55^*^ |
|  | SD | 0.98 | 1.00 | 0.85 | 1.17 |  | 0.91 | 1.03 | 1.12 | 0.93 |  |
| 16:1n-7 | mean | -0.10 | 0.04 | -0.14 | 0.20 | 0.09^*^ | -0.09 | 0.05 | -0.07 | 0.11 | 0.47^*^ |
|  | SD | 0.97 | 1.01 | 1.02 | 0.98 |  | 0.98 | 1.00 | 1.01 | 1.01 |  |
| 18:0 | mean | -0.01 | -0.11 | 0.15 | -0.03 | 0.33^*^ | -0.06 | 0.001 | 0.17 | -0.11 | 0.27^*^ |
|  | SD | 0.84 | 1.23 | 0.96 | 0.93 |  | 0.86 | 1.01 | 1.15 | 0.95 |  |
| 18:1n-9 | mean | -0.31^†‡^ | 0.22 | -0.15^§^ | 0.24 | <0.001^*^ | -0.15 | 0.11 | 0.06 | -0.02 | 0.34^*^ |
|  | SD | 0.91 | 1.11 | 0.94 | 0.94 |  | 0.89 | 1.07 | 1.07 | 0.95 |  |
| 18:1n-7 | mean | -0.11 | 0.11 | -0.14 | 0.15 | 0.12^*^ | -0.11 | 0.11 | -0.06 | 0.05 | 0.44^*^ |
|  | SD | 0.97 | 1.20 | 0.88 | 0.90 |  | 0.89 | 1.20 | 1.02 | 0.85 |  |
| 20:0 | mean | 0.08 | -0.01 | -0.17 | 0.11 | 0.25^*^ | 0.07 | 0.02 | -0.10 | 0.01 | 0.73^*^ |
|  | SD | 0.95 | 0.97 | 0.99 | 1.08 |  | 0.80 | 0.97 | 1.24 | 0.95 |  |
| 20:1n-9 | mean | -0.23^†^ | 0.24 | -0.06 | 0.05 | 0.01^*^ | -0.18 | 0.20 | 0.02 | -0.03 | 0.08^*^ |
|  | SD | 1.04 | 0.93 | 1.04 | 0.94 |  | 1.01 | 1.02 | 0.99 | 0.96 |  |
| 22:0 | mean | -0.12 | 0.18 | -0.12 | 0.07 | 0.12^*^ | -0.14 | 0.16 | 0.002 | -0.03 | 0.24^*^ |
|  | SD | 0.93 | 1.05 | 0.87 | 1.13 |  | 1.09 | 0.93 | 0.90 | 1.06 |  |
| 24:0 | mean | -0.44^\|\|¶^ | 0.52 | -0.37^\|\|¶^ | 0.30 | <0.001^*^ | -0.34^\|\|¶^ | 0.38 | -0.19^\|\|¶^ | 0.15 | <0.001^*^ |
|  | SD | 0.88 | 1.03 | 0.76 | 0.96 |  | 1.06 | 0.77 | 1.00 | 0.99 |  |
| 24:1n-9 | mean | -0.26^†^ | 0.26 | -0.10 | 0.11 | 0.002^*^ | -0.26^†^ | 0.25 | -0.05 | 0.06 | 0.01^*^ |
|  | SD | 1.08 | 0.88 | 0.84 | 1.11 |  | 1.06 | 0.90 | 0.90 | 1.08 |  |
|  | SD | 0.99 | 0.58 | 0.92 | 0.54 |  | 0.90 | 0.91 | 0.97 | 0.84 |  |
| Total | mean | -0.50^**^ | -0.81 | -0.61 | -0.54 | 0.04^*^ | -0.06 | 0.05 | 0.11 | -0.11 | 0.47^*^ |
|  | SD | 0.72 | 0.73 | 0.82 | 0.88 |  | 0.89 | 1.04 | 1.12 | 0.93 |  |

^*^One-way ANOVA followed by Tukey’s post-hoc test

significantly different from probiotics p<0.05^**^, p<0.01^†^, p<0.001^||^

significantly different from placebo p<0.05^§^, p<0.01^‡^, p<0.001^¶^

The following variables were natural log-transformed: 16:1n-7 %, 18:1n-7 %, 20:1n-9 %, 24:1n-9 %, 14:0 μg/mL, 16:1n-7 μg/mL, 18:1n-7 μg/mL, 20:0 μg/mL, 20:1n-9 μg/mL, 22:0 μg/mL, 24:0 μg/mL

Supplementary Table 7. Fatty acids in serum non-esterified fatty acids (NEFAs) as % of total fatty acids and absolute concentration (μg/mL) z-scores in pregnant women with overweight and obesity according to the four dietary intervention groups in late pregnancy. Data are expressed as mean and SD.

|  |  | NEFA fatty acids % of total (z-scores) | | | | | NEFA fatty acids absolute (μg/mL) (z-scores) | | | | |
| --- | --- | --- | --- | --- | --- | --- | --- | --- | --- | --- | --- |
|  |  | **Fish oil + placebo** | **Probiotics + placebo** | **Fish oil + probiotics** | **Placebo + placebo** | **P value** | **Fish oil + placebo** | **Probiotics + placebo** | **Fish oil + probiotics** | **Placebo + placebo** | **P value** |
| n |  | 90 | 92 | 91 | 88 |  | 90 | 92 | 91 | 88 |  |
| 14:0 | mean | -0.06 | 0.03 | -0.01 | 0.05 | 0.90^*^ | -0.03 | -0.01 | -0.02 | 0.07 | 0.91^*^ |
|  | SD | 0.99 | 0.84 | 1.18 | 0.97 |  | 1.00 | 0.83 | 1.18 | 0.97 |  |
| 16:0 | mean | -0.02 | 0.01 | -0.04 | 0.06 | 0.92^*^ | 0.02 | -0.09 | -0.04 | 0.11 | 0.60^*^ |
|  | SD | 0.91 | 1.01 | 0.90 | 1.17 |  | 1.05 | 0.94 | 1.06 | 0.94 |  |
| 16:1n-7 | mean | -0.07 | 0.001 | 0.02 | 0.05 | 0.85^*^ | -0.01 | -0.06 | -0.04 | 0.10 | 0.71^*^ |
|  | SD | 0.90 | 1.02 | 1.07 | 1.02 |  | 0.95 | 1.02 | 1.12 | 0.90 |  |
| 18:0 | mean | 0.04 | -0.10 | 0.02 | 0.05 | 0.71^*^ | 0.05 | -0.17 | -0.02 | 0.14 | 0.19^*^ |
|  | SD | 0.78 | 1.27 | 1.00 | 0.88 |  | 0.95 | 1.08 | 1.03 | 0.92 |  |
| 18:1n-9 | mean | 0.01 | 0.05 | -0.12 | 0.05 | 0.62^*^ | 0.03 | -0.06 | -0.04 | 0.07 | 0.82^*^ |
|  | SD | 0.93 | 1.01 | 1.09 | 0.97 |  | 1.05 | 1.00 | 1.06 | 0.90 |  |
| 18:1n-7 | mean | -0.02 | 0.06 | 0.08 | -0.12 | 0.54^*^ | -0.01 | 0.03 | 0.06 | -0.08 | 0.78^*^ |
|  | SD | 1.03 | 0.94 | 0.95 | 1.08 |  | 1.01 | 0.90 | 0.96 | 1.13 |  |
| 20:0 | mean | 0.12 | -0.12 | -0.12 | 0.12 | 0.15^*^ | 0.11 | -0.16 | -0.10 | 0.16 | 0.09^*^ |
|  | SD | 0.63 | 1.01 | 1.46 | 0.62 |  | 0.84 | 0.96 | 1.32 | 0.76 |  |
| 20:1n-9 | mean | -0.02 | -0.04 | -0.17 | 0.23 | 0.05^*^ | 0.002 | -0.10 | -0.15 | 0.26 | 0.04^†^ |
|  | SD | 0.86 | 1.00 | 1.14 | 0.96 |  | 0.86 | 0.88 | 1.23 | 0.96 |  |
| 22:0 | mean | 0.04 | -0.09 | -0.03 | 0.09 | 0.64^*^ | 0.05 | -0.12 | -0.03 | 0.11 | 0.45^*^ |
|  | SD | 0.97 | 1.11 | 0.97 | 0.94 |  | 0.98 | 1.09 | 0.98 | 0.94 |  |
| 24:0 | mean | -0.05 | 0.05 | -0.14 | 0.15 | 0.25^*^ | -0.04 | 0.04 | -0.14 | 0.15 | 0.26^*^ |
|  | SD | 1.03 | 0.96 | 1.04 | 0.97 |  | 1.04 | 0.94 | 1.04 | 0.97 |  |
| 24:1n-9 | mean | -0.01 | -0.04 | 0.04 | 0.01 | 0.94^*^ | -0.01 | -0.05 | 0.03 | 0.03 | 0.92^*^ |
|  | SD | 1.00 | 1.07 | 0.95 | 0.99 |  | 1.00 | 1.06 | 0.95 | 1.00 |  |
| Total | mean | -0.06 | -0.17 | 0.04 | -0.02 | 0.55^*^ | 0.02 | -0.09 | -0.03 | 0.10 | 0.61^*^ |
|  | SD | 0.89 | 1.07 | 0.95 | 1.10 |  | 1.04 | 0.94 | 1.12 | 0.90 |  |

^*^One-way ANOVA or ^†^Welch ANOVA followed by Tamhanes T’2 post-hoc test

The following variables were natural log-transformed: 14:0 %, 18:0 %, 18:1n-7 %, 20:0 %, 20:1n-9 %, 22:0 %, 24:0 %, 24:1n-9 %, 14:0 μg/mL, 16:0 μg/mL, 16:1n7 μg/mL, 18:0 μg/mL, 18:1n7 μg/mL, 20:0 μg/mL, 20:1n-9 μg/mL, 22:0 μg/mL, 24:0 μg/mL, 24:1n-9 μg/mL

Supplementary Table 8. Fatty acids in serum cholesterol esters (CEs) as % of total fatty acids and absolute concentration (μg/mL) z-scores in pregnant women with overweight and obesity according to the four dietary intervention groups in late pregnancy. Data are expressed as mean and SD.

|  | | CE fatty acids % of total (z-scores) | | | | |  | | CE fatty acids absolute concentration (μg/mL) (z-scores) | | | | |
| --- | --- | --- | --- | --- | --- | --- | --- | --- | --- | --- | --- | --- | --- |
|  |  | | **Fish oil + placebo** | **Probiotics + placebo** | **Fish oil + probiotics** | **Placebo + placebo** | | **P value** | **Fish oil + placebo** | **Probiotics + placebo** | **Fish oil + probiotics** | **Placebo + placebo** | **P value** |
| n |  | | 90 | 92 | 91 | 88 | |  | 90 | 92 | 91 | 88 |  |
| 14:0 | mean | | 0.004 | -0.04 | 0.03 | 0.01 | | 0.97^*^ | 0.01 | 0.001 | 0.06 | -0.08 | 0.85^*^ |
|  | SD | | 0.89 | 1.02 | 1.05 | 1.05 | |  | 1.02 | 1.01 | 0.94 | 1.03 |  |
| 16:0 | mean | | -0.12 | -0.04 | 0.10 | 0.07 | | 0.44^*^ | -0.05 | 0.01 | 0.11 | -0.08 | 0.60^*^ |
|  | SD | | 1.54 | 0.62 | 0.75 | 0.84 | |  | 1.23 | 0.89 | 0.92 | 0.93 |  |
| 16:1n-7 | mean | | -0.11 | -0.05 | 0.01 | 0.15 | | 0.35^*^ | -0.07 | -0.02 | 0.05 | 0.04 | 0.84^*^ |
|  | SD | | 1.14 | 0.98 | 0.95 | 0.91 | |  | 1.17 | 0.93 | 0.93 | 0.97 |  |
| 18:0 | mean | | 0.05 | -0.09 | 0.06 | -0.02 | | 0.73^*^ | 0.05 | -0.03 | 0.04 | -0.06 | 0.84^*^ |
|  | SD | | 0.95 | 0.70 | 1.33 | 0.94 | |  | 1.04 | 0.85 | 1.12 | 0.99 |  |
| 18:1n-9 | mean | | -0.12 | 0.15 | 0.03 | -0.07 | | 0.31^*^ | -0.04 | 0.10 | 0.02 | -0.08 | 0.63^*^ |
|  | SD | | 0.98 | 0.63 | 1.27 | 1.01 | |  | 1.20 | 0.65 | 1.06 | 1.02 |  |
| 18:1n-7 | mean | | -0.08 | -0.04 | -0.02 | 0.15 | | 0.44^*^ | -0.09 | 0.001 | 0.01 | 0.08 | 0.73^*^ |
|  | SD | | 1.16 | 1.01 | 0.87 | 0.93 | |  | 1.14 | 1.05 | 0.93 | 0.86 |  |
| 20:0 | mean | | 0.01 | 0.01 | -0.05 | 0.03 | | 0.96^*^ | 0.04 | 0.01 | 0.002 | -0.06 | 0.93^*^ |
|  | SD | | 0.95 | 0.92 | 1.02 | 1.11 | |  | 1.07 | 0.98 | 0.93 | 1.03 |  |
|  |  | |  |  |  |  | |  |  |  |  |  |  |
| 20:1n-9 | mean | | 0.03 | -0.02 | -0.06 | 0.05 | | 0.90^*^ | 0.02 | -0.02 | -0.04 | 0.04 | 0.96^*^ |
|  | SD | | 0.99 | 0.99 | 1.01 | 1.02 | |  | 1.00 | 0.98 | 1.01 | 1.03 |  |
| 22:0 | mean | | -0.07 | 0.04 | 0.002 | 0.04 | | 0.86^*^ | -0.06 | 0.05 | -0.002 | 0.02 | 0.90^*^ |
|  | SD | | 0.97 | 1.03 | 0.96 | 1.06 | |  | 0.97 | 1.04 | 0.96 | 1.05 |  |
| 24:0 | mean | | -0.01 | 0.13 | -0.05 | -0.07 | | 0.52^*^ | -0.01 | 0.14 | -0.04 | -0.09 | 0.46^*^ |
|  | SD | | 1.02 | 1.00 | 0.99 | 1.00 | |  | 1.02 | 1.00 | 1.00 | 0.98 |  |
| 24:1n-9 | mean | | -0.04 | -0.05 | 0.06 | 0.04 | | 0.85^*^ | -0.04 | -0.04 | 0.05 | 0.03 | 0.89^*^ |
|  | SD | | 1.00 | 0.99 | 1.01 | 1.02 | |  | 1.01 | 1.00 | 1.01 | 0.99 |  |
| Total | mean | | 0.14 | -0.07 | -0.09 | 0 | | 0.42^*^ | 0.02 | 0.04 | 0.05 | -0.12 | 0.66^*^ |
|  | SD | | 1.03 | 0.93 | 1.03 | 1.03 | |  | 1.04 | 0.85 | 1.05 | 1.06 |  |

^*^One-way ANOVA followed by Tukey’s post-hoc test

The following variables were log-transformed: 14:0 %, 16:0 %, 16:1n-7 %, 18:0 %, 18:1n7 %, 20:1n-9 %, 22:0 %, 24:0 %, 24:1n9 %, 14:0 μg/mL, 16:0 μg/mL, 16:1n-7 μg/mL, 18:0 μg/mL, 18:1n-9 μg/mL, 18:1n-7 μg/mL, 20:1n-9 μg/mL, 22:0 μg/mL, 24:0 μg/mL, 24:1n-9 μg/mL

Supplementary Table 9. Fatty acids in serum triacylglycerols (TAGs) as % of total fatty acids and absolute concentration (μg/mL) z-scores in pregnant women with overweight and obesity according to the four dietary intervention groups in late pregnancy. Data are expressed as mean and SD.

|  |  | TAG fatty acids % of total (z-scores) | | | | | TAG fatty acids absolute concentration (μg/mL) (z-scores) | | | | |
| --- | --- | --- | --- | --- | --- | --- | --- | --- | --- | --- | --- |
|  |  | **Fish oil + placebo** | **Probiotics + placebo** | **Fish oil + probiotics** | **Placebo + placebo** | **P value** | **Fish oil + placebo** | **Probiotics + placebo** | **Fish oil + probiotics** | **Placebo + placebo** | **P value** |
| n |  | 89 | 92 | 91 | 87 |  | 89 | 92 | 91 | 87 |  |
| 14:0 | mean | 0.08 | -0.01 | -0.05 | -0.01 | 0.85^*^ | 0.08 | 0.01 | -0.08 | -0.01 | 0.78^*^ |
|  | SD | 1.08 | 0.93 | 0.98 | 1.02 |  | 0.93 | 1.05 | 1.10 | 0.90 |  |
| 16:0 | mean | 0.04 | -0.07 | -0.10 | 0.14 | 0.36^*^ | 0.01 | 0.003 | 0.001 | -0.02 | 1.00^*^ |
|  | SD | 0.89 | 0.88 | 1.21 | 0.97 |  | 0.95 | 1.07 | 1.12 | 0.85 |  |
| 16:1n-7 | mean | -0.20 | 0.10 | -0.09 | 0.18 | 0.045^*^ | -0.09 | 0.09 | -0.10 | 0.11 | 0.34^*^ |
|  | SD | 0.86 | 0.94 | 0.96 | 1.18 |  | 1.02 | 0.94 | 1.12 | 0.90 |  |
| 18:0 | mean | 0.29^†‡^ | -0.16 | 0.002 | -0.13 | 0.01^*^ | 0.15 | -0.06 | -0.04 | -0.04 | 0.46^*^ |
|  | SD | 1.14 | 0.81 | 1.05 | 0.92 |  | 0.95 | 0.97 | 1.19 | 0.85 |  |
| 18:1n-9 | mean | -0.15^†§^ | 0.26 | -0.28^\|\|¶^ | 0.18 | <0.001^*^ | 0.01 | 0.04 | -0.11 | 0.05 | 0.69^*^ |
|  | SD | 0.85 | 0.84 | 1.20 | 0.98 |  | 0.92 | 1.04 | 1.17 | 0.85 |  |
| 18:1n-7 | mean | -0.20 | 0.01 | 0.14 | 0.05 | 0.13^*^ | -0.13 | 0.004 | 0.08 | 0.05 | 0.49^*^ |
|  | SD | 0.88 | 0.94 | 1.24 | 0.88 |  | 1.00 | 0.99 | 1.13 | 0.86 |  |
| 20:0 | mean | 0.23 | -0.15 | 0.01 | -0.09 | 0.06^*^ | 0.16 | -0.07 | -0.04 | -0.05 | 0.39^*^ |
|  | SD | 1.10 | 0.75 | 1.03 | 1.05 |  | 0.93 | 1.01 | 1.16 | 0.88 |  |
| 20:1n-9 | mean | -0.18 | 0.15 | -0.10 | 0.13 | 0.06^**^ | -0.04 | 0.06 | -0.08 | 0.06 | 0.72^*^ |
|  | SD | 0.86 | 1.18 | 0.85 | 1.04 |  | 0.88 | 1.04 | 1.05 | 1.03 |  |
| 22:0 | mean | -0.01 | -0.01 | -0.09 | 0.11 | 0.61^*^ | 0.06 | -0.07 | -0.08 | 0.08 | 0.59^*^ |
|  | SD | 0.94 | 0.98 | 0.96 | 1.12 |  | 0.94 | 1.05 | 1.05 | 0.96 |  |
| 24:0 | mean | -0.03 | 0.17 | -0.16 | 0.02 | 0.17^*^ | 0.02 | 0.13 | -0.13 | -0.03 | 0.36^*^ |
|  | SD | 0.97 | 1.02 | 0.96 | 1.04 |  | 0.94 | 1.00 | 1.00 | 1.06 |  |
| 24:1n-9 | mean | -0.11 | 0.06 | 0.01 | 0.03 | 0.68^*^ | -0.10 | 0.05 | 0.01 | 0.03 | 0.76^*^ |
|  | SD | 1.06 | 0.99 | 0.99 | 0.96 |  | 1.07 | 0.98 | 1.01 | 0.95 |  |
| Total | mean | -0.01 | -0.04 | 0.16 | -0.08 | 0.40^*^ | 0.02 | 0.02 | 0.004 | -0.04 | 0.98^*^ |
|  | SD | 0.86 | 1.03 | 1.05 | 1.08 |  | 0.98 | 1.08 | 1.09 | 0.84 |  |

^*^One-way ANOVA followed by Tukey’s post-hoc test or ^**^Welch ANOVA followed by Tamhanes T’2 post-hoc test

significantly different from probiotics p<0.05^†^, p<0.01^||^

significantly different from placebo p<0.05^‡^, p<0.01^¶^

^§^After adjusting for smoking before pregnancy the difference between fish oil and probiotics in the proportion of 18:1n-9 did not remain statistically significant.

The following variables were natural log-transformed: 18:1n-7 %, 24:1n-9 %, 14:0 μg/mL, 16:1n-7 μg/mL, 18:0 μg/mL, 18:1n-9 μg/mL, 18:1n-7 μg/mL, 20:0 μg/mL, 20:1n-9 μg/mL, 22:0 μg/mL, 24:0 μg/mL

Supplementary Table 10. The raw % values of all fatty acids in serum phosphatidylcholine (PC) in pregnant women with overweight and obesity according to the four dietary intervention groups in late pregnancy. Data are expressed as mean and SD or median and IQR.

|  |  | Fish oil + placebo | Probiotics + placebo | Fish oil + probiotics | Placebo + placebo |
| --- | --- | --- | --- | --- | --- |
| n |  | 90 | 91 | 91 | 88 |
| 14:0 | mean | 0.34 | 0.34 | 0.33 | 0.35 |
|  | SD | 0.10 | 0.10 | 0.09 | 0.11 |
| 16:0 | mean | 34.83 | 34.73 | 34.79 | 34.94 |
|  | SD | 1.43 | 1.47 | 1.24 | 1.71 |
| 16:1n-7 | median | 0.39 | 0.40 | 0.38 | 0.46 |
|  | IQR | 0.28-0.54 | 0.31-0.57 | 0.28-0.52 | 0.34-0.59 |
| 18:0 | mean | 10.04 | 9.92 | 10.22 | 10.01 |
|  | SD | 0.93 | 1.36 | 1.06 | 1.03 |
| 18:1n-9 | mean | 11.71 | 12.34 | 11.90 | 12.35 |
|  | SD | 1.07 | 1.31 | 1.11 | 1.11 |
| 18:1n-7 | median | 1.80 | 1.97 | 1.79 | 2.00 |
|  | IQR | 1.47-2.40 | 1.56-2.47 | 1.53-2.33 | 1.61-2.47 |
| 18:2n-6 | mean | 20.37 | 20.96 | 20.01 | 20.78 |
|  | SD | 2.18 | 2.67 | 1.88 | 2.45 |
| 18:3n-6 | median | 0.05 | 0.05 | 0.04 | 0.05 |
|  | IQR | 0.02-0.07 | 0.03-0.08 | 0.03-0.06 | 0.03-0.07 |
| 18:3n-3 | median | 0.04 | 0.05 | 0.04 | 0.05 |
|  | IQR | 0.03-0.07 | 0.03-0.08 | 0.03-0.07 | 0.03-0.08 |
| 20:0 | mean | 0.47 | 0.45 | 0.43 | 0.47 |
|  | SD | 0.14 | 0.14 | 0.14 | 0.16 |
| 20:1n-9 | median | 0.15 | 0.17 | 0.16 | 0.16 |
|  | IQR | 0.13-0.18 | 0.14-0.19 | 0.14-0.18 | 0.14-0.19 |
| 20:2n-6 | mean | 0.34 | 0.40 | 0.35 | 0.39 |
|  | SD | 0.09 | 0.11 | 0.09 | 0.12 |
| 20:3n-6 | mean | 3.33 | 3.81 | 3.32 | 3.71 |
|  | SD | 0.68 | 0.82 | 0.70 | 0.60 |
| 20:4n-6 | mean | 6.77 | 7.21 | 6.92 | 7.23 |
|  | SD | 1.27 | 1.28 | 1.46 | 1.54 |
| 22:0 | mean | 0.91 | 1.12 | 0.91 | 1.04 |
|  | SD | 0.66 | 0.75 | 0.62 | 0.81 |
| 20:4n-3 | median | 0.11 | 0.09 | 0.11 | 0.10 |
|  | IQR | 0.04-0.22 | 0.05-0.21 | 0.04-0.20 | 0.05-0.19 |
| 20:5n-3 | median | 1.26 | 0.50 | 1.04 | 0.53 |
|  | IQR | 0.79-1.52 | 0.40-0.63 | 0.80-1.69 | 0.37-0.75 |
| 24:0 | mean | 0.17 | 0.25 | 0.18 | 0.23 |
|  | SD | 0.07 | 0.08 | 0.06 | 0.08 |
| 24:1n-9 | median | 0.15 | 0.20 | 0.15 | 0.19 |
|  | IQR | 0.10-0.19 | 0.13-0.26 | 0.12-0.21 | 0.12-0.25 |
| 22:5n-3 | mean | 0.48 | 0.49 | 0.47 | 0.49 |
|  | SD | 0.14 | 0.12 | 0.10 | 0.12 |
| 22:6n-3 | mean | 6.07 | 4.08 | 6.14 | 4.19 |
|  | SD | 1.55 | 0.91 | 1.44 | 0.86 |

Supplementary Table 11. The raw % values of all fatty acids in serum non-esterified fatty acids (NEFAs) in pregnant women with overweight and obesity according to the four dietary intervention groups in late pregnancy. Data are expressed as mean and SD or median and IQR.

|  |  | Fish oil + placebo | Probiotics + placebo | Fish oil + probiotics | Placebo + placebo |
| --- | --- | --- | --- | --- | --- |
| n |  | 90 | 92 | 91 | 88 |
| 14:0 | median | 1.16 | 1.13 | 1.16 | 1.20 |
|  | IQR | 0.79-1.47 | 0.85-1.51 | 0.86-1.55 | 0.92-1.60 |
| 16:0 | mean | 25.31 | 25.40 | 25.26 | 25.56 |
|  | SD | 2.73 | 3.05 | 2.72 | 3.53 |
| 16:1n-7 | mean | 1.92 | 2.00 | 2.01 | 2.05 |
|  | SD | 0.85 | 0.96 | 1.01 | 0.97 |
| 18:0 | median | 14.64 | 14.78 | 14.78 | 14.72 |
|  | IQR | 12.83-16.86 | 12.63-16.58 | 12.51-17.14 | 12.78-16.81 |
| 18:1n-9 | mean | 30.55 | 30.80 | 29.74 | 30.82 |
|  | SD | 5.79 | 6.27 | 6.79 | 6.07 |
| 18:1n-7 | median | 4.48 | 5.31 | 5.21 | 4.19 |
|  | IQR | 1.98-8.52 | 2.08-7.77 | 1.98-7.81 | 2.00-7.07 |
| 18:2n-6 | mean | 10.29 | 10.29 | 10.16 | 10.08 |
|  | SD | 1.47 | 1.70 | 1.46 | 1.75 |
| 18:3n-6 | median | 0.09 | 0.08 | 0.07 | 0.10 |
|  | IQR | 0-0.16 | 0-0.15 | 0.00-0.14 | 0-0.23 |
| 18:3n-3 | median | 0.07 | 0.08 | 0.07 | 0.09 |
|  | IQR | 0-0.15 | 0-0.13 | 0-0.18 | 0-0.20 |
| 20:0 | median | 1.42 | 1.36 | 1.39 | 1.38 |
|  | IQR | 1.22-1.79 | 1.14-1.60 | 1.11-1.63 | 1.19-1.62 |
| 20:1n-9 | median | 0.32 | 0.32 | 0.32 | 0.38 |
|  | IQR | 0.23-0.43 | 0.23-0.42 | 0.23-0.39 | 0.28-0.48 |
| 20:2n-6 | median | 0.14 | 0.11 | 0.14 | 0.14 |
|  | IQR | 0-0.40 | 0-0.29 | 0-0.32 | 0-0.89 |
| 20:3n-6 | median | 0.44 | 0.47 | 0.43 | 0.50 |
|  | IQR | 0.30-0.65 | 0.34-0.69 | 0.30-0.69 | 0.32-0.76 |
| 20:4n-6 | mean | 2.34 | 2.64 | 2.40 | 2.46 |
|  | SD | 0.80 | 0.88 | 0.88 | 0.86 |
| 22:0 | median | 0.30 | 0.28 | 0.21 | 0.28 |
|  | IQR | 0.12-0.42 | 0.10-0.46 | 0.10-0.44 | 0.13-0.52 |
| 20:4n-3 | median | 0.20 | 0.18 | 0.20 | 0.25 |
|  | IQR | 0.10-0.46 | 0.03-0.38 | 0.07-0.46 | 0.06-0.45 |
| 20:5n-3 | median | 0.29 | 0.19 | 0.28 | 0.21 |
|  | IQR | 0.19-0.46 | 0.11-0.29 | 0.16-0.41 | 0.13-0.35 |
| 24:0 | median | 0.09 | 0.11 | 0.08 | 0.11 |
|  | IQR | 0-0.21 | 0-0.18 | 0-0.17 | 0.01-0.24 |
| 24:1n-9 | median | 0.35 | 0.42 | 0.35 | 0.38 |
|  | IQR | 0.19-0.63 | 0.13-0.69 | 0.22-0.74 | 0.24-0.65 |
| 22:5n-3 | median | 0.21 | 0.23 | 0.22 | 0.21 |
|  | IQR | 0.14-0.29 | 0.16-0.33 | 0.14-0.31 | 0.13-0.27 |
| 22:6n-3 | median | 2.62 | 1.63 | 2.44 | 1.65 |
|  | IQR | 1.94-3.21 | 1.17-2.22 | 2.00-3.47 | 1.23-1.93 |

Supplementary Table 12. The raw % values of all fatty acids in serum cholesteryl esters (CEs) in pregnant women with overweight and obesity according to the four dietary intervention groups in late pregnancy. Data are expressed as mean and SD or median and IQR.

|  |  | Fish oil + placebo | Probiotics + placebo | Fish oil + probiotics | Placebo + placebo |
| --- | --- | --- | --- | --- | --- |
| n |  | 90 | 92 | 91 | 88 |
| 14:0 | median | 0.65 | 0.63 | 0.63 | 0.65 |
|  | IQR | 0.49-0.81 | 0.45-0.82 | 0.48-0.87 | 0.44-0.86 |
| 16:0 | median | 12.62 | 12.57 | 12.56 | 12.60 |
|  | IQR | 12.01-13.25 | 11.79-13.09 | 12.09-13.33 | 11.71-13.32 |
| 16:1n-7 | median | 2.51 | 2.53 | 2.44 | 2.87 |
|  | IQR | 1.70-3.45 | 1.81-3.41 | 1.85-3.42 | 1.89-3.78 |
| 18:0 | median | 0.61 | 0.63 | 0.62 | 0.61 |
|  | IQR | 0.54-0.74 | 0.55-0.71 | 0.53-0.75 | 0.54-0.72 |
| 18:1n-9 | mean | 22.30 | 23.17 | 22.78 | 22.46 |
|  | SD | 3.30 | 2.11 | 4.27 | 3.38 |
| 18:1n-7 | median | 1.87 | 2.06 | 1.99 | 2.54 |
|  | IQR | 1.32-3.38 | 1.42-3.29 | 1.48-3.13 | 1.46-3.61 |
| 18:2n-6 | mean | 48.21 | 48.35 | 47.52 | 47.48 |
|  | SD | 5.91 | 5.84 | 7.44 | 6.57 |
| 18:3n-6 | median | 0.20 | 0.29 | 0.20 | 0.28 |
|  | IQR | 0.03-0.35 | 0.02-0.40 | 0.02-0.30 | 0.03-0.40 |
| 18:3n-3 | median | 0.18 | 0.16 | 0.16 | 0.20 |
|  | IQR | 0.12-0.38 | 0.11-0.36 | 0.10-0.39 | 0.11-0.44 |
| 20:0 | mean | 0.99 | 0.99 | 0.98 | 1.00 |
|  | SD | 0.26 | 0.25 | 0.28 | 0.31 |
| 20:1n-9 | median | 0.06 | 0.06 | 0.06 | 0.06 |
|  | IQR | 0.03-0.11 | 0.02-0.12 | 0.02-0.10 | 0.02-0.10 |
| 20:2n-6 | median | 0.04 | 0.04 | 0.04 | 0.05 |
|  | IQR | 0.02-0.07 | 0.03-0.08 | 0.02-0.07 | 0.02-0.07 |
| 20:3n-6 | median | 0.68 | 0.75 | 0.68 | 0.72 |
|  | IQR | 0.58-0.77 | 0.63-0.85 | 0.57-0.74 | 0.61-0.83 |
| 20:4n-6 | mean | 4.67 | 4.73 | 4.66 | 4.72 |
|  | SD | 1.14 | 1.29 | 1.33 | 1.41 |
| 22:0 | median | 0.53 | 0.68 | 0.65 | 0.72 |
|  | IQR | 0.20-0.87 | 0.24-0.98 | 0.21-0.93 | 0.23-1.02 |
| 20:4n-3 | median | 0.09 | 0.07 | 0.08 | 0.08 |
|  | IQR | 0.06-0.14 | 0.03-0.16 | 0.05-0.13 | 0.04-0.16 |
| 20:5n-3 | median | 0.88 | 0.35 | 0.78 | 0.37 |
|  | IQR | 0.49-1.25 | 0.26-0.47 | 0.51-1.02 | 0.24-0.51 |
| 24:0 | median | 0 | 0 | 0 | 0 |
|  | IQR | 0-0.08 | 0-0.09 | 0-0.05 | 0-0.07 |
| 24:1n-9 | median | 0 | 0 | 0.01 | 0.02 |
|  | IQR | 0-0.09 | 0-0.10 | 0-0.11 | 0-0.11 |
| 22:5n-3 | median | 0.14 | 0.14 | 0.14 | 0.14 |
|  | IQR | 0.06-0.24 | 0.05-0.28 | 0.07-0.31 | 0.07-0.25 |
| 22:6n-3 | median | 0.60 | 0.36 | 0.57 | 0.38 |
|  | IQR | 0.38-0.79 | 0.24-0.44 | 0.43-0.73 | 0.30-0.48 |

Supplementary Table 13. The raw % values of all fatty acids in serum triacylglycerols (TAGs) in pregnant women with overweight and obesity according to the four dietary intervention groups in late pregnancy. Data are expressed as mean and SD or median and IQR.

|  |  | Fish oil + placebo | Probiotics + placebo | Fish oil + probiotics | Placebo + placebo |
| --- | --- | --- | --- | --- | --- |
| n |  | 89 | 92 | 91 | 87 |
| 14:0 | mean | 2.26 | 2.19 | 2.16 | 2.19 |
|  | SD | 0.81 | 0.70 | 0.74 | 0.77 |
| 16:0 | mean | 31.11 | 30.69 | 30.54 | 31.52 |
|  | SD | 3.58 | 3.53 | 4.85 | 3.88 |
| 16:1n-7 | mean | 2.73 | 3.05 | 2.85 | 3.13 |
|  | SD | 0.90 | 0.99 | 1.01 | 1.24 |
| 18:0 | mean | 3.26 | 2.94 | 3.05 | 2.96 |
|  | SD | 0.81 | 0.58 | 0.74 | 0.65 |
| 18:1n-9 | mean | 38.46 | 40.04 | 37.93 | 39.74 |
|  | SD | 3.33 | 3.31 | 4.71 | 3.83 |
| 18:1n-7 | median | 2.46 | 2.62 | 2.71 | 2.72 |
|  | IQR | 2.08-3.09 | 2.28-3.29 | 2.16-3.93 | 2.33-3.43 |
| 18:2n-6 | median | 12.65 | 12.62 | 12.87 | 12.03 |
|  | IQR | 11.29-14.45 | 11.19-14.18 | 11.58-14.31 | 10.84-13.62 |
| 18:3n-6 | median | 0.09 | 0.09 | 0.08 | 0.09 |
|  | IQR | 0.06-0.11 | 0.07-0.11 | 0.06-0.11 | 0.07-0.11 |
| 18:3n-3 | median | 0.14 | 0.17 | 0.14 | 0.14 |
|  | IQR | 0.10-0.19 | 0.13-0.22 | 0.09-0.2 | 0.11-0.22 |
| 20:0 | mean | 1.51 | 1.35 | 1.41 | 1.37 |
|  | SD | 0.46 | 0.31 | 0.43 | 0.44 |
| 20:1n-9 | mean | 0.33 | 0.37 | 0.34 | 0.37 |
|  | SD | 0.11 | 0.15 | 0.11 | 0.14 |
| 20:2n-6 | median | 0.09 | 0.12 | 0.12 | 0.13 |
|  | IQR | 0.07-0.13 | 0.08-0.17 | 0.08-0.16 | 0.10-0.18 |
| 20:3n-6 | mean | 0.23 | 0.27 | 0.25 | 0.25 |
|  | SD | 0.08 | 0.09 | 0.11 | 0.09 |
| 20:4n-6 | median | 1.17 | 1.23 | 1.16 | 1.12 |
|  | IQR | 0.87-1.53 | 0.89-1.55 | 0.92-1.56 | 0.84-1.45 |
| 22:0 | mean | 0.17 | 0.17 | 0.16 | 0.19 |
|  | SD | 0.13 | 0.13 | 0.13 | 0.15 |
| 20:4n-3 | median | 0.06 | 0.05 | 0.06 | 0.05 |
|  | IQR | 0.04-0.09 | 0.03-0.07 | 0.03-0.08 | 0.03-0.08 |
| 20:5n-3 | median | 0.34 | 0.13 | 0.28 | 0.12 |
|  | IQR | 0.17-0.54 | 0.09-0.19 | 0.18-0.43 | 0.07-0.22 |
| 24:0 | mean | 0.07 | 0.08 | 0.07 | 0.07 |
|  | SD | 0.05 | 0.05 | 0.04 | 0.05 |
| 24:1n-9 | median | 0.05 | 0.05 | 0.05 | 0.05 |
|  | IQR | 0-0.09 | 0-0.10 | 0-0.09 | 0-0.08 |
| 22:5n-3 | mean | 0.14 | 0.10 | 0.14 | 0.10 |
|  | SD | 0.07 | 0.05 | 0.07 | 0.07 |
| 22:6n-3 | median | 1.75 | 0.67 | 1.76 | 0.59 |
|  | IQR | 1.12-2.43 | 0.50-0.94 | 0.95-2.40 | 0.43-0.88 |

Supplementary Table 14. Fatty acids in serum phosphatidylcholine (PC) which differed statistically significantly in late pregnancy between the four dietary intervention groups adjusted with their early pregnancy values. Fatty acids are expressed as % of total fatty acids and absolute concentration (μg/mL) z-scores. Data are expressed as mean and SE.

|  | | | PC fatty acids % of total (z-scores) | | | | | PC fatty acids absolute concentration (μg/mL) (z-scores) | | | | |
| --- | --- | --- | --- | --- | --- | --- | --- | --- | --- | --- | --- | --- |
|  |  | **Fish oil + placebo** | | **Probiotics + placebo** | **Fish oil + probiotics** | **Placebo + placebo** | **P value^*^** | **Fish oil + placebo** | **Probiotics + placebo** | **Fish oil + probiotics** | **Placebo + placebo** | **P value^*^** |
|  | n | 88 | | 91 | 90 | 86 |  | 88 | 91 | 90 | 86 |  |
| 20:5n-3 | mean | 0.63^\|\|¶^ | | -0.63 | 0.59^\|\|¶^ | -0.64 | <0.001 | 0.49^\|\|¶^ | -0.48 | 0.53^\|\|¶^ | -0.58 | <0.001 |
|  | SE | 0.08 | | 0.08 | 0.08 | 0.08 |  | 0.08 | 0.08 | 0.08 | 0.08 |  |
| 22:6n-3 | mean | 0.59^\|\|¶^ | | -0.65 | 0.65^\|\|¶^ | -0.63 | <0.001 | .37^\|\|¶^ | -0.40 | 0.44^\|\|¶^ | -0.43 | <0.001 |
|  | SE | 0.07 | | 0.07 | 0.07 | 0.07 |  | 0.07^†^ | 0.07 | 0.07 | 0.07 |  |
| 18:2n-6 | mean | -0.05 | | 0.20 | -0.23^†^ | 0.08 | 0.008 |  |  |  |  |  |
|  | SE | 0.09 | | 0.09 | 0.09 | 0.10 |  |  |  |  |  |  |
| 20:2n-6 | mean | -0.25^†§^ | | 0.26 | -0.17^§^ | 0.19 | <0.001 | -0.23^†^ | 0.25 | -0.06 | 0.07 | 0.003 |
|  | SE | 0.10 | | 0.10 | 0.10 | 0.10 |  | 0.10 | 0.09 | 0.09 | 0.10 |  |
| 20:3n-6 | mean | -0.33^\|\|¶^ | | 0.35 | -0.29^\|\|¶^ | 0.26 | <0.001 | -0.25^\|\|‡^ | 0.28 | -0.13^\|\|^ | 0.10 | <0.001 |
|  | SE | 0.08 | | 0.08 | 0.08 | 0.08 |  | 0.07 | 0.07 | 0.07 | 0.08 |  |
| 18:1n-9 | mean | -0.29^\|\|¶^ | | 0.22 | -0.23^†¶^ | 0.33 | <0.001 |  |  |  |  |  |
|  | SE | 0.09 | | 0.09 | 0.09 | 0.09 |  |  |  |  |  |  |
| 20:1n-9 | mean | -0.23^†^ | | 0.23 | -0.05 | 0.03 | <0.001 |  |  |  |  |  |
|  | SE | 0.10 | | 0.10 | 0.10 | 0.10 |  |  |  |  |  |  |
| 24:0 | mean | -0.41^\|\|¶^ | | 0.48 | -0.36^\|\|¶^ | 0.29 | <0.001 | -0.30^\|\|‡^ | 0.35 | -0.21^\|\|§^ | 0.17 | <0.001 |
|  | SE | 0.09 | | 0.09 | 0.09 | 0.09 |  | 0.09 | 0.09 | 0.09 | 0.09 |  |
| 24:1n-9 | mean | -0.19^**^ | | 0.17 | -0.10 | 0.10 | 0.03 | -0.19^**^ | 0.17 | -0.06 | 0.07 | 0.04 |
|  | SE | 0.09 | | 0.09 | 0.09 | 0.10 |  | 0.09 | 0.09 | 0.09 | 0.09 |  |
| Total n-3 LC-PUFAs | mean | 0.62^\|\|¶^ | | -0.64 | 0.62^\|\|¶^ | -0.64 | <0.001 | 0.36^\|\|¶^ | -0.38 | 0.44^\|\|¶^ | -0.44 | <0.001 |
|  | SE | 0.07 | | 0.07 | 0.07 | 0.07 |  | 0.07 | 0.07 | 0.07 | 0.07 |  |
| Total n-6 LC-PUFAs | mean | -0.25^\|\|‡^ | | 0.37 | -0.34^\|\|¶^ | 0.24 | <0.001 |  |  |  |  |  |
|  | SE | 0.10 | | 0.09 | 0.09 | 0.10 |  |  |  |  |  |  |
| n-6/n-3 LC-PUFA ratio | mean |  | |  |  |  |  | -0.55^\|\|¶^ | 0.61 | -0.59^\|\|¶^ | 0.57 | <0.001 |
|  | SE |  | |  |  |  |  | 0.07 | 0.07 | 0.07 | 0.08 |  |
| Total | mean | -0.52 | | -0.81 | -0.60 | -0.54 | 0.05 |  |  |  |  |  |
|  | SE | 0.08 | | 0.08 | 0.08 | 0.09 |  |  |  |  |  |  |

^*^General linear model followed by Bonferroni

significantly different from probiotics p<0.05^**^, p<0.01^†^, p<0.001^||^

significantly different from placebo p<0.05^§^, p<0.01^‡^, p<0.001^¶^

Supplementary Table 15. Fatty acids in serum non-esterified fatty acids (NEFAs) which differed statistically significantly in late pregnancy between the four dietary intervention groups adjusted with their early pregnancy values. Fatty acids are expressed as % of total fatty acids and absolute concentration (μg/mL) z-scores. Data are expressed as mean and SE.

|  |  | NEFA fatty acids % of total (z-scores) | | | | | NEFA fatty acids absolute concentration (μg/mL) (z-scores) | | | | |
| --- | --- | --- | --- | --- | --- | --- | --- | --- | --- | --- | --- |
|  |  | **Fish oil + placebo** | **Probiotics + placebo** | **Fish oil + probiotics** | **Placebo + placebo** | **P value^*^** | **Fish oil + placebo** | **Probiotics + placebo** | **Fish oil + probiotics** | **Placebo + placebo** | **P value^*^** |
|  | n | 88 | 92 | 91 | 86 |  | 88 | 92 | 91 | 86 |  |
| 20:5n-3 | mean | 0.22^**^ | -0.23 | 0.05 | -0.07 | 0.016 | 0.23^†^ | -0.25 | 0.06 | -0.06 | 0.009 |
|  | SE | 0.10 | 0.10 | 0.10 | 0.10 |  | 0.10 | 0.10 | 0.10 | 0.10 |  |
| 22:6n-3 | mean | 0.34^\|\|¶^ | -0.26 | 0.31^\|\|¶^ | -0.45 | <0.001 | 0.33^\|\|¶^ | -0.23 | 0.24^\|\|¶^ | -0.39 | <0.001 |
|  | SE | 0.08 | 0.08 | 0.08 | 0.09 |  | 0.08 | 0.08 | 0.08 | 0.08 |  |
| 20:1n-9 | mean |  |  |  |  |  | -0.01 | -0.07 | -0.13 | 0.22 | 0.09 |
|  | SE |  |  |  |  |  | 0.10 | 0.10 | 0.10 | 0.10 |  |
| Total n-3 LC-PUFAs | mean | 0.23^†¶^ | -0.26 | 0.35^\|\|¶^ | -0.38 | <0.001 | 0.21^†¶^ | -0.24 | 0.29^\|\|¶^ | -0.29 | <0.001 |
|  | SE | 0.09 | 0.09 | 0.09 | 0.09 |  | 0.09 | 0.09 | 0.09 | 0.09 |  |
| n-6/n-3 LC-PUFA ratio | mean |  |  |  |  |  | -0.29^\|\|¶^ | 0.32 | -0.37^\|\|¶^ | 0.40 | <0.001 |
|  | SE |  |  |  |  |  | 0.09 | 0.09 | 0.09 | 0.10 |  |

^*^General linear model followed by Bonferroni

significantly different from probiotics p<0.05^**^, p<0.01^†^, p<0.001^||^

significantly different from placebo p<0.05^§^, p<0.01^‡^, p<0.001^¶^

Supplementary Table 16. Fatty acids in serum cholesteryl esters (CEs) which differed statistically significantly in late pregnancy between the four dietary intervention groups adjusted with their early pregnancy values. Fatty acids are expressed as % of total fatty acids and absolute concentration (μg/mL) z-scores. Data are expressed as mean and SE.

|  |  | CE fatty acids % of total (z-scores) | | | | | CE fatty acids absolute concentration (μg/mL) (z-scores) | | | | |
| --- | --- | --- | --- | --- | --- | --- | --- | --- | --- | --- | --- |
|  |  | **Fish oil + placebo** | **Probiotics + placebo** | **Fish oil + probiotics** | **Placebo + placebo** | **P value^*^** | **Fish oil + placebo** | **Probiotics + placebo** | **Fish oil + probiotics** | **Placebo + placebo** | **P value^*^** |
|  | n | 88 | 92 | 91 | 86 |  | 88 | 92 | 91 | 86 |  |
| 20:5n-3 | mean | 0.48^\|\|¶^ | -0.42 | 0.45^\|\|¶^ | -0.54 | <0.001 | 0.38^\|\|¶^ | -0.32 | 0.40^\|\|¶^ | -0.49 | <0.001 |
|  | SE | 0.08 | 0.08 | 0.08 | 0.08 |  | 0.08 | 0.08 | 0.08 | 0.08 |  |
| 22:6n-3 | mean | 0.21^\|\|§^ | -0.33 | 0.32^\|\|¶^ | -0.22 | <0.001 | 0.17^†§^ | -0.29 | 0.33^\|\|¶^ | -0.23 | <0.001 |
|  | SE | 0.10 | 0.09 | 0.09 | 0.10 |  | 0.09 | 0.09 | 0.09 | 0.09 |  |
| 20:3n-6 | mean | -0.19^†§^ | 0.21 | -0.17^†§^ | 0.15 | <0.001 |  |  |  |  |  |
|  | SE | 0.08 | 0.08 | 0.08 | 0.08 |  |  |  |  |  |  |
| Total n-3 LC-PUFAs | mean | 0.38^\|\|¶^ | -0.44 | 0.45^\|\|¶^ | -0.40 | <0.001 | 0.27 | -0.27 | 0.28 | -0.29 | <0.001 |
|  | SE | 0.08 | 0.07 | 0.07 | 0.08 |  | 0.07 | 0.07 | 0.07 | 0.07 |  |
|  | n |  |  |  |  |  | 88 | 91 | 91 | 86 |  |
| n-6/n-3 LC-PUFA ratio | mean |  |  |  |  |  | -0.34^\|\|¶^ | 0.45 | -0.42^\|\|¶^ | 0.34 | <0.001 |
|  | SE |  |  |  |  |  | 0.08 | 0.08 | 0.08 | 0.08 |  |

^*^General linear model followed by Bonferroni

significantly different from probiotics p<0.05^**^, p<0.01^†^, p<0.001^||^

significantly different from placebo p<0.05^§^, p<0.01^‡^, p<0.001^¶^

Supplementary Table 17. Fatty acids in serum triacylfglycerols (TAGs) which differed statistically significantly in late pregnancy between the four dietary intervention groups adjusted with their early pregnancy values. Fatty acids are expressed as % of total fatty acids and absolute concentration (μg/mL) z-scores. Data are expressed as mean and SE.

|  |  | TAG fatty acids % of total (z-scores) | | | | | TAG fatty acids absolute concentration (μg/mL) (z-scores) | | | | |
| --- | --- | --- | --- | --- | --- | --- | --- | --- | --- | --- | --- |
|  |  | **Fish oil + placebo** | **Probiotics + placebo** | **Fish oil + probiotics** | **Placebo + placebo** | **P value^*^** | **Fish oil + placebo** | **Probiotics + placebo** | **Fish oil + probiotics** | **Placebo + placebo** | **P value^*^** |
|  | n | 87 | 92 | 91 | 85 |  | 87 | 92 | 91 | 85 |  |
| 20:5n-3 | mean | 0.52^\|\|¶^ | -0.37 | 0.38^\|\|¶^ | -0.56 | <0.001 | 0.50^\|\|¶^ | -0.34 | 0.32^\|\|¶^ | -0.50 | <0.001 |
|  | SE | 0.09 | 0.09 | 0.09 | 0.09 |  | 0.08 | 0.08 | 0.08 | 0.09 |  |
| 22:5n-3 | mean | 0.21^†‡^ | -0.29 | 0.30^\|\|¶^ | -0.27 | <0.001 | 0.12 | -0.13 | 0.17^§^ | -0.19 | 0.01 |
|  | SE | 0.10 | 0.10 | 0.10 | 0.10 |  | 0.09 | 0.09 | 0.09 | 0.09 |  |
| 22:6n-3 | mean | 0.64^\|\|¶^ | -0.52 | 0.57^\|\|¶^ | -0.73 | <0.001 | 0.54^\|\|¶^ | -0.43 | 0.43^\|\|¶^ | -0.57 | <0.001 |
|  | SE | 0.08 | 0.08 | 0.08 | 0.08 |  | 0.08 | 0.08 | 0.08 | 0.08 |  |
| 20:2n-6 | mean | -0.20^§^ | 0.12 | -0.09 | 0.17 | 0.02 | -0.13 | 0.12 | -0.14 | 0.16 | 0.03 |
|  | SE | 0.10 | 0.09 | 0.10 | 0.10 |  | 0.09 | 0.09 | 0.09 | 0.09 |  |
| 18:0 | mean | 0.32^†§^ | -0.15 | -0.01 | -0.15 | 0.005 |  |  |  |  |  |
|  | SE | 0.10 | 0.10 | 0.10 | 0.10 |  |  |  |  |  |  |
| 18:1n-9 | mean | -0.14 | 0.22 | -0.25^†^ | 0.17 | 0.002 |  |  |  |  |  |
|  | SE | 0.10 | 0.10 | 0.10 | 0.10 |  |  |  |  |  |  |
| Total n-3 LC-PUFAs | mean | 0.60^\|\|¶^ | -0.49 | 0.50^\|\|¶^ | -0.64 | <0.001 | 0.48^\|\|¶^ | -0.39 | 0.34^\|\|¶^ | -0.45 | <0.001 |
|  | SE | 0.08 | 0.08 | 0.08 | 0.08 |  | 0.09 | 0.08 | 0.08 | 0.09 |  |
|  | n |  |  |  |  |  | 87 | 92 | 90 | 85 |  |
| n-6/n-3 LC-PUFA ratio | mean |  |  |  |  |  | -0.62^\|\|¶^ | 0.53 | -0.52^\|\|¶^ | 0.64 | <0.001 |
|  | SE |  |  |  |  |  | 0.08 | 0.08 | 0.08 | 0.08 |  |

^*^General linear model followed by Bonferroni

significantly different from probiotics p<0.05^**^, p<0.01^†^, p<0.001^||^

significantly different from placebo p<0.05^§^, p<0.01^‡^, p<0.001^¶^

Supplementary Table 18. n-3 long-chain fatty acids (LC-PUFAs) in serum phosphatidylcholine (PC) in women receiving fish oil or probiotics and women receiving no fish oil or no probiotics (fish oil vs no-fish oil, probiotics vs no-probiotics) in late pregnancy. Data are expressed as mean and SE and mean difference and SE.

|  |  | PC fatty acids % of total (z-scores) | | | | | | PC fatty acids absolute concentration (μg/mL) (z-scores) | | | | | |
| --- | --- | --- | --- | --- | --- | --- | --- | --- | --- | --- | --- | --- | --- |
|  |  | Mean | SE | Mean difference^*^ | SE^*^ | P value | Interaction P value | Mean | SE | Mean difference^*^ | SE^*^ | P value | Interaction P value |
| 18:3n-3 | No fish oil | 0.05 | 0.08 | -0.09 | 0.11 | 0.40 | 0.28 | 0.03 | 0.08 | -0.07 | 0.11 | 0.52 | 0.26 |
|  | Fish oil | -0.04 | 0.07 |  |  |  |  | -0.03 | 0.07 |  |  |  |  |
|  | No probiotics | -0.02 | 0.08 | 0.04 | 0.11 | 0.73 |  | -0.03 | 0.08 | 0.06 | 0.11 | 0.55 |  |
|  | Probiotics | 0.02 | 0.07 |  |  |  |  | 0.03 | 0.07 |  |  |  |  |
| 20:4n-3 | No fish oil | 0.01 | 0.08 | -0.02 | 0.11 | 0.83 | 0.64 | 0.00 | 0.08 | -0.01 | 0.11 | 0.95 | 0.68 |
|  | Fish oil | -0.01 | 0.08 |  |  |  |  | 0.00 | 0.08 |  |  |  |  |
|  | No probiotics | 0.01 | 0.08 | -0.02 | 0.11 | 0.86 |  | 0.00 | 0.08 | 0.00 | 0.11 | 1.00 |  |
|  | Probiotics | -0.01 | 0.07 |  |  |  |  | 0.00 | 0.07 |  |  |  |  |
| 20:5n-3 | No fish oil | -0.63 | 0.06 | 1.26 | 0.08 | <0.001 | 0.96 | -0.55 | 0.06 | 1.09 | 0.09 | <0.001 | 0.93 |
|  | Fish oil | 0.63 | 0.06 |  |  |  |  | 0.54 | 0.06 |  |  |  |  |
|  | No probiotics | 0.04 | 0.06 | -0.09 | 0.08 | 0.30 |  | 0.01 | 0.06 | -0.02 | 0.09 | 0.81 |  |
|  | Probiotics | -0.05 | 0.06 |  |  |  |  | -0.01 | 0.06 |  |  |  |  |
| 22:5n-3 | No fish oil | 0.08 | 0.08 | -0.15 | 0.11 | 0.15 | 0.44 | 0.02 | 0.08 | -0.04 | 0.11 | 0.68 | 0.66 |
|  | Fish oil | -0.08 | 0.07 |  |  |  |  | -0.02 | 0.08 |  |  |  |  |
|  | No probiotics | 0.02 | 0.08 | -0.05 | 0.11 | 0.66 |  | -0.04 | 0.08 | 0.08 | 0.11 | 0.45 |  |
|  | Probiotics | -0.02 | 0.07 |  |  |  |  | 0.04 | 0.07 |  |  |  |  |
| 22:6n-3 | No fish oil | -0.63 | 0.06 | 1.25 | 0.08 | <0.001 | 0.49 | -0.44 | 0.07 | 0.86 | 0.10 | <0.001 | 0.65 |
|  | Fish oil | 0.62 | 0.06 |  |  |  |  | 0.43 | 0.07 |  |  |  |  |
|  | No probiotics | 0.00 | 0.06 | -0.01 | 0.08 | 0.86 |  | -0.03 | 0.07 | -0.01 | 0.08 | 0.55 |  |
|  | Probiotics | -0.01 | 0.06 |  |  |  |  | 0.03 | 0.07 |  |  |  |  |
| Total n-3 LC-PUFAs | No fish oil | -0.63 | 0.06 | 1.26 | 0.08 | <0.001 | 0.77 | -0.44 | 0.07 | 0.87 | 0.10 | <0.001 | 0.78 |
|  | Fish oil | 0.63 | 0.06 |  |  |  |  | 0.43 | 0.07 |  |  |  |  |
|  | No probiotics | 0.03 | 0.06 | -0.06 | 0.08 | 0.50 |  | -0.02 | 0.07 | 0.04 | 0.10 | 0.67 |  |
|  | Probiotics | -0.03 | 0.06 |  |  |  |  | 0.02 | 0.07 |  |  |  |  |
| n-6/n-3 LC-PUFA ratio | No fish oil | |  |  |  |  |  | 0.59 | 0.06 | -1.17 | 0.09 | <0.001 | 0.35 |
|  | Fish oil |  |  |  |  |  |  | -0.58 | 0.06 |  |  |  |  |
|  | No probiotics | |  |  |  |  |  | -0.02 | 0.06 | 0.04 | 0.09 | 0.62 |  |
|  | Probiotics | |  |  |  |  |  | 0.02 | 0.06 |  |  |  |  |

^*^Mean difference and SE are for fish oil vs no-fish oil or probiotics vs no-probiotics

Supplementary Table 19. n-3 long-chain fatty acids (LC-PUFAs) in serum non-esterified fatty acids (NEFAs) in women receiving fish oil or probiotics and women receiving no fish oil or no probiotics (fish oil vs no-fish oil, probiotics vs no-probiotics) in late pregnancy. Data are expressed as mean and SE and mean difference and SE.

|  |  | NEFA fatty acids % of total (z-scores) | | | | | | NEFA fatty acids absolute concentration (μg/mL) (z-scores) | | | | | |
| --- | --- | --- | --- | --- | --- | --- | --- | --- | --- | --- | --- | --- | --- |
|  |  | Mean | SE | Mean difference^*^ | SE^*^ | P value | Interaction P value | Mean | SE | Mean difference^*^ | SE^*^ | P value | Interaction P value |
| 18:3n-3 | No fish oil | 0.03 | 0.07 | -0.06 | 0.11 | 0.56 | 0.11 | 0.02 | 0.08 | -0.03 | 0.11 | 0.75 | 0.12 |
|  | Fish oil | -0.03 | 0.07 |  |  |  |  | -0.02 | 0.07 |  |  |  |  |
|  | No probiotics | 0.05 | 0.08 | -0.09 | 0.11 | 0.37 |  | 0.05 | 0.08 | -0.10 | 0.11 | 0.34 |  |
|  | Probiotics | -0.05 | 0.07 |  |  |  |  | -0.05 | 0.07 |  |  |  |  |
| 20:4n-3 | No fish oil | -0.06 | 0.08 | 0.12 | 0.11 | 0.27 | 0.79 | -0.06 | 0.08 | 0.12 | 0.11 | 0.25 | 0.70 |
|  | Fish oil | 0.06 | 0.07 |  |  |  |  | 0.06 | 0.07 |  |  |  |  |
|  | No probiotics | 0.06 | 0.08 | -0.13 | 0.11 | 0.23 |  | 0.07 | 0.08 | -0.14 | 0.11 | 0.18 |  |
|  | Probiotics | -0.06 | 0.07 |  |  |  |  | -0.07 | 0.07 |  |  |  |  |
| 20:5n-3 | No fish oil | -0.15 | 0.07 | 0.30 | 0.10 | 0.004 | 0.83 | -0.15 | 0.07 | 0.31 | 0.10 | 0.003 | 1.00 |
|  | Fish oil | 0.15 | 0.07 |  |  |  |  | 0.16 | 0.07 |  |  |  |  |
|  | No probiotics | 0.11 | 0.07 | -0.22 | 0.10 | 0.03 |  | 0.13 | 0.07 | -0.25 | 0.10 | 0.02 |  |
|  | Probiotics | -0.11 | 0.07 |  |  |  |  | -0.12 | 0.07 |  |  |  |  |
| 22:5n-3 | No fish oil | 0.03 | 0.08 | -0.07 | 0.11 | 0.52 | 0.27 | 0.03 | 0.08 | -0.06 | 0.11 | 0.59 | 0.34 |
|  | Fish oil | -0.04 | 0.07 |  |  |  |  | -0.03 | 0.08 |  |  |  |  |
|  | No probiotics | -0.02 | 0.08 | 0.03 | 0.11 | 0.76 |  | 0.01 | 0.08 | -0.02 | 0.11 | 0.86 |  |
|  | Probiotics | 0.02 | 0.07 |  |  |  |  | -0.01 | 0.07 |  |  |  |  |
| 22:6n-3 | No fish oil | -0.35 | 0.07 | 0.70 | 0.10 | <0.001 | 0.71 | 0.03 | 0.08 | -0.06 | 0.11 | 0.59 | 0.34 |
|  | Fish oil | 0.35 | 0.07 |  |  |  |  | -0.03 | 0.08 |  |  |  |  |
|  | No probiotics | -0.04 | 0.07 | 0.08 | 0.10 | 0.45 |  | 0.01 | 0.08 | -0.02 | 0.11 | 0.86 |  |
|  | Probiotics | 0.04 | 0.07 |  |  |  |  | -0.01 | 0.07 |  |  |  |  |
| Total n-3 LC-PUFAs | No fish oil | -0.33 | 0.07 | 0.66 | 0.10 | <0.001 | 0.89 | -0.28 | 0.07 | 0.56 | 0.10 | <0.001 | 0.56 |
|  | Fish oil | 0.33 | 0.07 |  |  |  |  | 0.28 | 0.07 |  |  |  |  |
|  | No probiotics | -0.02 | 0.07 | 0.03 | 0.10 | 0.78 |  | 0.03 | 0.07 | -0.06 | 0.10 | 0.58 |  |
|  | Probiotics | 0.01 | 0.07 |  |  |  |  | -0.03 | 0.07 |  |  |  |  |
| n-6/n-3 LC-PUFA ratio | No fish oil | |  |  |  |  |  | 0.36 | 0.07 | -0.72 | 0.10 | <0.001 | 0.86 |
|  | Fish oil |  |  |  |  |  |  | -0.36 | 0.07 |  |  |  |  |
|  | No probiotics | |  |  |  |  |  | 0.01 | 0.07 | -0.01 | 0.10 | 0.89 |  |
|  | Probiotics | |  |  |  |  |  | -0.01 | 0.07 |  |  |  |  |

^*^Mean difference and SE are for fish oil vs no-fish oil or probiotics vs no-probiotics

Supplementary Table 20. n-3 long-chain fatty acids (LC-PUFAs) in serum cholesteryl esters (CEs) in women receiving fish oil or probiotics and women receiving no fish oil or no probiotics (fish oil vs no-fish oil, probiotics vs no-probiotics) in late pregnancy. Data are expressed as mean and SE and mean difference and SE.

|  |  | CE fatty acids % of total (z-scores) | | | | | | | | | | | CE fatty acids absolute concentration (μg/mL) (z-scores) | | | | | | |
| --- | --- | --- | --- | --- | --- | --- | --- | --- | --- | --- | --- | --- | --- | --- | --- | --- | --- | --- | --- |
|  |  | Mean | | SE | | Mean difference^*^ | SE^*^ | | P value | | Interaction P value | | Mean | SE | Mean difference^*^ | | SE^*^ | P value | Interaction P value |
| 18:3n-3 | No fish oil | -0.03 | | 0.08 | | 0.06 | 0.11 | | 0.60 | | 0.76 | | -0.04 | 0.08 | 0.07 | | 0.11 | 0.49 | 0.58 |
|  | Fish oil | 0.03 | | 0.08 | |  |  | |  | |  | | 0.04 | 0.08 |  | |  |  |  |
|  | No probiotics | 0.05 | | 0.08 | | -0.10 | 0.11 | | 0.33 | |  | | 0.03 | 0.08 | -0.06 | | 0.11 | 0.58 |  |
|  | Probiotics | -0.05 | | 0.07 | |  |  | |  | |  | | -0.03 | 0.07 |  | |  |  |  |
| 20:4n-3 | No fish oil | -0.07 | | 0.08 | | 0.15 | 0.11 | | 0.17 | | 0.58 | | -0.08 | 0.07 | 0.17 | | 0.11 | 0.12 | 0.39 |
|  | Fish oil | 0.07 | | 0.07 | |  |  | |  | |  | | 0.08 | 0.07 |  | |  |  |  |
|  | No probiotics | 0.05 | | 0.08 | | -0.11 | 0.11 | | 0.32 | |  | | 0.04 | 0.08 | -0.07 | | 0.11 | 0.49 |  |
|  | Probiotics | -0.05 | | 0.07 | |  |  | |  | |  | | -0.04 | 0.07 |  | |  |  |  |
| 20:5n-3 | No fish oil | -0.49 | | 0.07 | | 0.97 | 0.09 | | <0.001 | | 0.40 | | -0.42 | 0.07 | 0.84 | | 0.10 | <0.001 | 0.38 |
|  | Fish oil | 0.48 | | 0.07 | |  |  | |  | |  | | 0.42 | 0.07 |  | |  |  |  |
|  | No probiotics | 0.01 | | 0.07 | | -0.03 | 0.09 | | 0.74 | |  | | 0.00 | 0.07 | 0.00 | | 0.10 | 0.99 |  |
|  | Probiotics | -0.02 | | 0.07 | |  |  | |  | |  | | 0.00 | 0.07 |  | |  |  |  |
| 22:5n-3 | No fish oil | 0.00 | | 0.08 | | 0.01 | 0.11 | | 0.96 | | 0.13 | | 0.00 | 0.08 | 0.01 | | 0.11 | 0.93 | 0.13 |
|  | Fish oil | 0.00 | | 0.07 | |  |  | |  | |  | | 0.01 | 0.07 |  | |  |  |  |
|  | No probiotics | -0.02 | | 0.08 | | 0.04 | 0.11 | | 0.70 | |  | | -0.03 | 0.08 | 0.06 | | 0.11 | 0.56 |  |
|  | Probiotics | 0.02 | | 0.07 | |  |  | |  | |  | | 0.03 | 0.07 |  | |  |  |  |
| 22:6n-3 | No fish oil | -0.30 | | 0.07 | | 0.60 | 0.10 | | <0.001 | | 0.67 | | -0.30 | 0.07 | 0.59 | | 0.10 | <0.001 | 0.76 |
|  | Fish oil | 0.30 | | 0.07 | |  |  | |  | |  | | 0.30 | 0.07 |  | |  |  |  |
|  | No probiotics | 0.03 | | 0.07 | | -0.06 | 0.10 | | 0.56 | |  | | 0.02 | 0.07 | -0.03 | | 0.10 | 0.76 |  |
|  | Probiotics | -0.03 | | 0.07 | |  |  | |  | |  | | -0.02 | 0.07 |  | |  |  |  |
| Total n-3 LC-PUFAs | No fish oil | -0.46 | | 0.07 | | 0.93 | 0.09 | | <0.001 | | 0.68 | | -0.33 | 0.07 | 0.65 | | 0.10 | <0.001 | 0.72 |
|  | Fish oil | 0.46 | | 0.07 | |  |  | |  | |  | | 0.33 | 0.07 |  | |  |  |  |
|  | No probiotics | 0.06 | | 0.07 | | -0.12 | 0.09 | | 0.19 | |  | | 0.06 | 0.07 | -0.12 | | 0.10 | 0.25 |  |
|  | Probiotics | -0.06 | | 0.07 | |  |  | |  | |  | | -0.06 | 0.07 |  | |  |  |  |
| n-6/n-3 LC-PUFA ratio | No fish oil | |  | |  | | |  | |  | |  | 0.43 | 0.07 | | -0.86 | 0.10 | <0.001 | 0.42 |
|  | Fish oil |  | |  | |  |  | |  | |  | | -0.43 | 0.07 |  | |  |  |  |
|  | No probiotics | |  | |  | | |  | |  | |  | -0.05 | 0.07 | | 0.10 | 0.10 | 0.32 |  |
|  | Probiotics | |  | |  | | |  | |  | |  | 0.05 | 0.07 | |  |  |  |  |

^*^Mean difference and SE are for fish oil vs no-fish oil or probiotics vs no-probiotics

Supplementary Table 21. n-3 long-chain fatty acids (LC-PUFAs) in serum triacylglycerols (TAGs) in women receiving fish oil or probiotics and women receiving no fish oil or no probiotics (fish oil vs no-fish oil, probiotics vs no-probiotics) in late pregnancy. Data are expressed as mean and SE and mean difference and SE.

|  |  | TAG fatty acids % of total (z-scores) | | | | | | | | | | TAG fatty acids absolute concentration (μg/mL) (z-scores) | | | | | | |
| --- | --- | --- | --- | --- | --- | --- | --- | --- | --- | --- | --- | --- | --- | --- | --- | --- | --- | --- |
|  |  | Mean | | SE | | Mean difference^*^ | SE^*^ | | P value | Interaction P value | | Mean | SE | Mean difference^*^ | | SE^*^ | P value | Interaction P value |
| 18:3n-3 | No fish oil | 0.12 | | 0.07 | | -0.24 | 0.11 | | 0.02 | 0.48 | | 0.10 | 0.08 | -0.21 | | 0.11 | 0.05 | 0.44 |
|  | Fish oil | -0.12 | | 0.07 | |  |  | |  |  | | -0.10 | 0.07 |  | |  |  |  |
|  | No probiotics | -0.06 | | 0.08 | | 0.12 | 0.11 | | 0.24 |  | | -0.04 | 0.08 | 0.07 | | 0.11 | 0.51 |  |
|  | Probiotics | 0.06 | | 0.07 | |  |  | |  |  | | 0.03 | 0.07 |  | |  |  |  |
| 20:4n-3 | No fish oil | -0.03 | | 0.08 | | 0.06 | 0.11 | | 0.60 | 0.34 | | -0.02 | 0.08 | 0.05 | | 0.11 | 0.65 | 0.27 |
|  | Fish oil | 0.03 | | 0.08 | |  |  | |  |  | | 0.03 | 0.08 |  | |  |  |  |
|  | No probiotics | 0.06 | | 0.08 | | -0.13 | 0.11 | | 0.24 |  | | 0.07 | 0.08 | -0.13 | | 0.11 | 0.23 |  |
|  | Probiotics | -0.06 | | 0.07 | |  |  | |  |  | | -0.06 | 0.07 |  | |  |  |  |
| 20:5n-3 | No fish oil | -0.45 | | 0.07 | | 0.90 | 0.09 | | <0.001 | 0.13 | | -0.44 | 0.07 | 0.88 | | 0.10 | <0.001 | 0.13 |
|  | Fish oil | 0.45 | | 0.07 | |  |  | |  |  | | 0.44 | 0.07 |  | |  |  |  |
|  | No probiotics | 0.01 | | 0.07 | | -0.03 | 0.09 | | 0.78 |  | | 0.03 | 0.07 | -0.05 | | 0.10 | 0.58 |  |
|  | Probiotics | -0.02 | | 0.07 | |  |  | |  |  | | -0.03 | 0.07 |  | |  |  |  |
| 22:5n-3 | No fish oil | -0.28 | | 0.07 | | 0.57 | 0.10 | | <0.001 | 0.93 | | -0.18 | 0.07 | 0.37 | | 0.10 | <0.001 | 0.74 |
|  | Fish oil | 0.28 | | 0.07 | |  |  | |  |  | | 0.18 | 0.07 |  | |  |  |  |
|  | No probiotics | 0.01 | | 0.07 | | -0.02 | 0.10 | | 0.85 |  | | -0.01 | 0.07 | 0.02 | | 0.10 | 0.88 |  |
|  | Probiotics | -0.01 | | 0.07 | |  |  | |  |  | | 0.01 | 0.07 |  | |  |  |  |
| 22:6n-3 | No fish oil | -0.62 | | 0.06 | | 1.23 | 0.08 | | <0.001 | 0.15 | | -0.53 | 0.06 | 1.06 | | 0.09 | <0.001 | 0.20 |
|  | Fish oil | 0.61 | | 0.06 | |  |  | |  |  | | 0.53 | 0.06 |  | |  |  |  |
|  | No probiotics | -0.02 | | 0.06 | | 0.04 | 0.08 | | 0.68 |  | | -0.01 | 0.06 | 0.01 | | 0.09 | 0.94 |  |
|  | Probiotics | 0.02 | | 0.06 | |  |  | |  |  | | 0.00 | 0.06 |  | |  |  |  |
| Total n-3 LC-PUFAs | No fish oil | -0.56 | | 0.06 | | 1.11 | 0.09 | | <0.001 | 0.18 | | -0.45 | 0.07 | 0.89 | | 0.10 | <0.001 | 0.30 |
|  | Fish oil | 0.55 | | 0.06 | |  |  | |  |  | | 0.45 | 0.07 |  | |  |  |  |
|  | No probiotics | 0.00 | | 0.06 | | -0.01 | 0.09 | | 0.91 |  | | 0.03 | 0.07 | -0.05 | | 0.10 | 0.58 |  |
|  | Probiotics | -0.01 | | 0.06 | |  |  | |  |  | | -0.03 | 0.07 |  | |  |  |  |
| n-6/n-3 LC-PUFA ratio | No fish oil | |  | |  | | |  | |  |  | 0.58 | 0.06 | | -1.17 | 0.09 | <0.001 | 0.31 |
|  | Fish oil |  | |  | |  |  | |  |  | | -0.58 | 0.06 |  | |  |  |  |
|  | No probiotics | |  | |  | | |  | |  |  | -0.01 | 0.06 | | 0.02 | 0.09 | 0.85 |  |
|  | Probiotics | |  | |  | | |  | |  |  | 0.01 | 0.06 | |  |  |  |  |

^*^Mean difference and SE are for fish oil vs no-fish oil or probiotics vs no-probiotics
